# Metabolomics profiling of the free and total oxidised lipids in urine by LC-MS/MS: application in patients with rheumatoid arthritis

**DOI:** 10.1007/s00216-016-9742-2

**Published:** 2016-07-12

**Authors:** Junzeng Fu, Johannes C. Schoeman, Amy C. Harms, Herman A. van Wietmarschen, Rob J. Vreeken, Ruud Berger, Bart V. J. Cuppen, Floris P. J. G. Lafeber, Jan van der Greef, Thomas Hankemeier

**Affiliations:** 1 Department of Analytical Biosciences, Leiden Academic Center for Drug Research, Leiden University, Einsteinweg 55, 2333 CC Leiden, The Netherlands; 2 Sino-Dutch Center for Preventive and Personalized Medicine, P.O. Box 360, 3700 AJ Zeist, The Netherlands; 3 Netherlands Metabolomics Centre, Leiden University, Einsteinweg 55, 2333 CC Leiden, The Netherlands; 4 TNO, Netherlands Organization for Applied Scientific Research, Microbiology and Systems Biology, P.O. Box 360, 3700 AJ Zeist, The Netherlands; 5 Discovery Sciences, Janssen R&D, Turnhoutseweg 30, 2340 Beerse, Belgium; 6 Rheumatology and Clinical Immunology, University Medical Center Utrecht, F02.127, Heidelberglaan 100, 3584 CX Utrecht, The Netherlands

**Keywords:** LC-MS/MS, Metabolomics, Oxidized lipids, Urine, β-glucuronidase

## Abstract

Oxidised lipids, covering enzymatic and auto-oxidation-synthesised mediators, are important signalling metabolites in inflammation while also providing a readout for oxidative stress, both of which are prominent physiological processes in a plethora of diseases. Excretion of these metabolites via urine is enhanced through the phase-II conjugation with glucuronic acid, resulting in increased hydrophilicity of these lipid mediators. Here, we developed a bovine liver-β-glucuronidase hydrolysing sample preparation method, using liquid chromatography coupled to tandem mass spectrometry to analyse the total urinary oxidised lipid profile including the prostaglandins, isoprostanes, dihydroxy-fatty acids, hydroxy-fatty acids and the nitro-fatty acids. Our method detected more than 70 oxidised lipids biosynthesised from two non-enzymatic and three enzymatic pathways in urine samples. The total oxidised lipid profiling method was developed and validated for human urine and was demonstrated for urine samples from patients with rheumatoid arthritis. Pro-inflammatory mediators PGF_2α_ and PGF_3α_ and oxidative stress markers iPF_2α_- IV, 11-HETE and 14-HDoHE were positively associated with improvement of disease activity score. Furthermore, the anti-inflammatory nitro-fatty acids were negatively associated with baseline disease activity. In conclusion, the developed methodology expands the current metabolic profiling of oxidised lipids in urine, and its application will enhance our understanding of the role these bioactive metabolites play in health and disease.

**Electronic supplementary material:**

The online version of this article (doi:10.1007/s00216-016-9742-2) contains supplementary material, which is available to authorized users.

## Introduction

Oxidised lipids are important signalling mediators in health and disease, capable of providing quantitative readouts relating to inflammatory and oxidative stress status. The de novo synthesis of oxidised lipids can be broadly divided into enzymatic and auto-oxidation pathways. The auto-oxidation pathway of oxidised lipids is interlinked with reactive oxygen species (ROS) or reactive nitrogen species (RNS), leading to the peroxidation of fatty acids in membrane-bound phospholipids and producing the isoprostanes (IsoPs) [1, 2] or nitro-fatty acids (NO_2_-FAs) [3]. The lipid peroxidation readout from IsoPs are considered the golden standard for measuring oxidative stress in biological systems [4, 5]. Interestingly, NO_2_-FAs potentiate diverse anti-inflammatory signalling actions regarded as beneficial within health and disease [3]. These peroxidised lipids impair membrane and organelle integrity and are subsequently excreted from the cell into systemic circulation via cellular repair mechanisms [1].

The enzymatic routes include: (i) cyclooxygenase-I/II(COX-I/II), synthesising the prostaglandins (PGs); (ii) 5-/12-/15-lipoxygenase (5-/12-/15-LOX), synthesising leukotrienes, lipoxins and hydroxyl-fatty acids; and lastly, (iii) cytochrome P450 (CYP450) responsible for the synthesis of epoxy-fatty acids and dihydroxy-fatty acids [6]. These enzymatically oxidised lipids are synthesised locally from essential free fatty acids and act as signalling mediators in immune modulation and inflammatory responses [6–9]. Due to the potent biological signalling activity of enzymatically oxidised lipids, the active mediators are short-lived in systemic circulation where they are actively metabolised prior to excretion [10]. Thus taking serum and/or plasma as a representative snapshot of the systemic circulation might not be the most suitable approach to study the oxidised lipid profile. Urine, on the other hand, is a non-invasive bio-fluid, which contains the collected excreted downstream metabolic products. These downstream metabolites provide an enriched systemic readout and are indicative of the presence of the active upstream mediators.

Urine does present some sample specific complications for analysis, such as rather large variations in metabolite concentration, limited solubilities of apolar metabolites and conjugation of some metabolites. These complexities are even more prominent during the analyses of lipid-like metabolites in urine. Due to the partial hydrophobic nature, oxidised lipids are often conjugated to increase their hydrophilicity, mainly by phase-II metabolism located in the liver [11]. Phase-II metabolism comprises different enzymatic conjugation reactions, with oxidised lipids most commonly conjugated with glucuronic acid (GlcA) via UDP-glucuronosyltransferases [12–18]. Effectively, the oxidised lipids can be excreted in different forms via urine: the unconjugated (free) and the conjugated species [14, 16]. Actually, the GlcA-conjugated oxidised lipids represent a more hydrophilic form of the metabolites.

Robust metabolic profiling of urinary oxidised lipids has been reported using either gas- or liquid chromatography coupled to mass spectrometry [4, 19–27]. However, these methods either detected metabolites in their free form (neglecting the conjugated forms) or focused on a subset of IsoPs and/or PGs. These methodologies lack the broad scope of compounds necessary for a more thorough understanding of disease pathophysiology. Two recent methods reporting on the total (free + conjugated) IsoPs and PGs levels showed increasing urinary metabolite concentrations ranging from 36 to 100 % [15, 28], indicating the significant increases when measuring the total concentration, but these methods excluded the LOX and CYP450-oxidised lipid metabolites. Therefore, it is necessary to develop a method able to measure the total urinary oxidised lipids covering the three enzymatic synthesis routes as well as the auto-oxidation metabolites, broadening its biological range.

In the present study, we developed and validated robust methods for measuring both the free and total levels of oxidised lipids in human urine samples, covering the PGs, IsoPs, hydroxyl-fatty acids, epoxy-fatty acids, leukotrienes, lipoxins and NO_2_-FAs. To measure the total oxidised urinary profile, we investigated the suitability of three different β-glucuronidase enzymes derived from *Helix pomatia*, *Escherichia coli* and bovine liver. We evaluated these by determining the increase in free metabolites, metabolite stability and enzyme blank effect. Bovine liver derived β-glucuronidase was chosen as the preferred hydrolysing agent and was used in the total oxidised lipid method and validated concurrently with the free oxidised lipid method. Furthermore, we evaluated the benefit of total level oxidised lipid analyses in urine of rheumatoid arthritis patients. The methodology we established covers a broad scope of oxidised lipid, which enables further investigation of the function and mechanism of these lipids in both health and disease.

## Materials and methods

### Chemicals and reagents

Ultra-performance liquid chromatography (UPLC)-grade acetonitrile, isopropanol, methanol, ethyl acetate and purified water were purchased from Biosolve B.V. (Valkenswaard, the Netherlands). Acetic acid, ammonium hydroxide, ammonium acetate and 2-proponal were acquired from Sigma-Aldrich (Zwijndrecht, the Netherlands). Sodium dihydrogen phosphate dihydrate, sodium hydrogen phosphate and sodium acetate were obtained from Merck (Darmstadt, Germany).

#### β-glucuronidase enzymes

β-glucuronidase (GUS) from (1) *H. pomatia* type H-2 (aqueous solution, ≥85,000 units/mL), (2) *E. coli* IX-A (lyophilised power, 1,000,000–5,000,000 units/g) and (3) bovine liver B-1 (solid, ≥1,000,000 units/g), together with the exogenous substrate of GUS 4-methylumbelliferyl β-D-glucopyranoside (MUG) were purchased from Sigma-Aldrich Corporation (St. Louis, MO, USA).

#### Standards and internal standard solutions

Standards and deuterated standards were purchased from Cayman Chemicals (Ann Arbor, MI, USA), Bio-mol (Plymouth Meeting, PA, USA) or Larodan (Malmö, Sweden). Standard and deuterated standard solutions were prepared in methanol containing butylated hydroxy-toluene (BHT) (0.2 mg BHT/EDTA), stored at −80 °C. Electronic supplementary material (ESM) Table S1 lists an overview of the deuterated internal standards (ISTDs) used in this study.

### Urine sample collection

#### Collection of control urine for method development

Morning urine was obtained from ten volunteers (five males and five females) age 27 to 32. The urine samples were pooled, mixed and 400 μL were aliquoted into 2 mL Eppendorf tubes and immediately stored at −80 °C prior to extraction.

#### Rheumatoid arthritis patients

Oxidised lipid profiling was performed on urine samples derived from an observational study—BiOCURA [29]. In BiOCURA, rheumatoid arthritis (RA) patients eligible for biological disease-modifying anti-rheumatic drugs (bDMARDs) were recruited, and urine samples were collected at random times as baseline samples before initiating bDMARD therapy. Clinical parameters and demographic data were also collected at the start of the study as baseline information, including disease activity measured in 28 joints (DAS28), C-reactive protein (CRP), age, sex, BMI, smoking status, alcohol consumption and concomitant DMARDs. The study was approved by the ethics committee of the University Medical Centre Utrecht and the institutional review boards of the participating centres. Human material and human data were handled in accordance with the Declaration of Helsinki, and written informed consent was obtained from each patient.

Our analysis was restricted to the BiOCURA patients with a baseline (before bDMARDs treatment) disease activity score of >2.6 and good or no drug response after 3 months with Etanercept (ETN) or Adalimumab (ADA) based on the EULAR response criteria. EULAR good response is defined as an improvement in DAS28 > 1.2 and a present DAS28 ≤ 3.2, whereas a EULAR non-response is assigned to patients with an improvement of 0.6–1.2 with present DAS28 > 5.1 or patients with an improvement ≤0.6. In the end, 40 subjects (20 good responders, 20 non-responders) with ETN and 40 subjects (20 good responders, 20 non-responders) with ADA were included in the present study. ETN and ADA are tumor necrosis factor (TNF)-α inhibitors, which is the most widely used category of bDMARDs.

### Methodology development for optimising enzymatic hydrolysis

Hydrolysis conditions were optimised for each of the three GUSs following the approach in ESM Fig. S1. Parameters included enzyme concentration (1000, 1500 and 2000 U/sample), incubation temperature (37 and 55 °C) and time (2, 6, 12 and 24 h). The obtained experimental results for the three enzymes were used to determine the optimal hydrolysing conditions.

#### Enzymatic hydrolysing conditions

Literature was used to guide the selection of the optimal hydrolysing conditions for the three selected candidate enzymes with regard to the used buffer composition and pH [18, 30, 31]. For both, *H. pomatia* and bovine liver GUS, a 200-mM acetate buffer (pH 4.5) was used for hydrolysis, and for *E. coli* GUS, a hydrolysis buffer of 75 mM phosphate buffer (pH 6.8) was used. As a sensitive GUS substrate, MUG was added into each urine sample as a positive control for monitoring GUS activity.

#### Enzymatic hydrolysis procedure for GlcA-conjugated oxidised lipids

Ice-thawed urine samples (400 μL each) were immediately treated with 10 μL antioxidants (0.2 mg BHT/EDTA) and spiked with 20 μL ISTDs and 5 μL MUG solution. Next, 200 μL of the specific enzyme solution in its appropriate buffer was added to the sample, and the mixture was vortexed and incubated. After hydrolysis, samples were put on ice prior to oxidised lipid extraction.

### Total and free urinary oxidised lipid extraction

For both, total and free oxidised lipids, the extraction was performed by the same ethyl acetate liquid-liquid extraction (LLE) procedure. For the analyses of the free oxidised lipids, 400 μL urine were spiked with 10 μL antioxidants and 20 μL ISTDs, similar to the description of enzymatic hydrolysed (total) samples in “Enzymatic hydrolysis procedure for GlcA-conjugated oxidised lipids”. Subsequently, 200 μL citric acid/phosphate buffer (pH 3) was added to the total and free urine samples, and oxidised lipids were extracted by adding 1 mL ethyl acetate followed by shaking for 1 min. Samples were centrifuged at 13,000 rpm for 10 min (4 °C), after which 800 μL upper organic phase was transferred to a new Eppendorf tube. The LLE was repeated for a second time, and the collected organic phase was evaporated to dryness in Labconco CentriVap concentrator (Kansas City, MO, USA). The residues were reconstituted with 30 μL solution of 70 % methanol solution containing 100 nM 1-cyclohexyluriedo-3-dodecanoic acid (CUDA) as an external quality marker for the analysis. Afterwards, the extracts were centrifuged at 13,000 rpm for 5 min (4 °C) and transferred to LC autosampler vials.

### Lipid chromatography-mass spectrometry analyses (LC-MS/MS)

The complete oxidised lipid target lists and corresponding ISTDs divided per pathway are shown in ESM Table S2, S3, S4 and S5. The leukotrienes, hydroxyl-fatty acids, epoxy-fatty acids and lipoxins were analysed by high-performance liquid chromatography (Agilent 1260, San Jose, CA, USA) coupled to a triple-quadrupole mass spectrometer (Agilent 6460, San Jose, CA, USA), using an Ascentis® Express column (2.7 μm, 2.1 × 150 mm) as detailed in Strassburg et al. [32].

For adequate PG and IsoP isomer resolution, together with sensitive detection of the NO_2_-FAs, an optimised chromatographic method was developed in-house. UHPLC-MS/MS analysis was performed using the Shimadzu LCMS-8050 (Shimadzu, Japan) with a Kromasil EternityXT column (1.8 μm, 50 × 2.1 mm) maintained at 40 °C. The method used a three mobile-phase setup with: H_2_O with 5 mM ammonium acetate and 0.0625 % ammonium hydroxide (A), methanol with 0.2 % ammonium hydroxide (B) and isopropanol with 0.2 % ammonium hydroxide (C), with a flow rate of 0.6 mL/min. The injection volume was 10 μL, and all analytes eluted during a 10-min ternary gradient with a starting percentage composition of 94.5:5:0.5 (A/B/C). A chromatographic gradient is provided in ESM Fig. S2.

The LCMS-8050 consisted of a triple-quadrupole mass spectrometer with a heated electrospray ionisation (ESI) source. In negative ion mode, the source parameters were as follows: the heat block temperature was 400 °C, with the heating gas at 250 °C and a flow of 10 L/min. The nebulising and drying gas had a flow rate of 3 and 10 L/min, respectively. The interface voltage was set at 4 kV with a temperature of 150 °C. The conversion dynode was set at 10 kV, and desolvation temperature was 250 °C. Analytes were detected in negative MRM mode.

### Method validation

The targeted profiling of oxidised lipids has previously been validated for plasma samples, and the performance characteristics linearity, intra- and inter-day precision and accuracy were reported [32]. In the present study, we used the same chromatography parameters in terms of the columns, mobile phase, gradient etc. Therefore, the current validation was performed to determine recovery, matrix effect and precision for the ISTDs for the reported extraction method.

### Creatinine analysis

Urine samples of RA patients were collected at a random time of day, therefore the amount of liquids consumed influenced the concentration of the oxidised lipids in the samples. To eliminate this influence, levels of urinary creatinine were used to correct for dilution. Creatinine levels were determined based on a fast creatinine (urinary) assay kit (item no. 500701, Cayman Chemical Company, Ann Arbor, MI, USA).

### Data processing and statistical analyses

Peak determination and peak area integration were performed with Mass Hunter Quantitative Analysis (Agilent, Version B.04.00) and LabSolutions (Shimadzu, Version 5.65). The obtained peak areas of targets were first corrected by appropriate ISTD and creatinine concentrations (mg/dL), then normalised by log transformation. After data pre-processing, categorical principal component analysis (CATPCA, ESM methods) [33] and multiple linear regressions (MLR) were applied to explore the relationships between oxidised lipids and clinical parameters. Cytoscape was used to visualise the associations [34]. All statistical analysis was performed using IBM SPSS Statistics 23.0 software (Chicago, IL, USA).

## Results

### Optimisation for the hydrolysis of IsoPs, PGs and NO_2_-FAs

For determining the optimal enzymatic deconjugation procedure for urinary oxidised lipids, three GUSs derived from *H. pomatia*, *E. coli* and bovine liver were investigated. During the method development, critical parameters including enzyme concentrations, hydrolysis temperature and time were optimised. In order to simplify the method optimisation, we focused on the quantification of a pre-selected panel of metabolites to evaluate the method performance, which covers the most studied IsoPs, PGs and NO_2_-FAs in human body fluids. The selected panel consisted of F-series IsoPs (8-iso-PGF_2α_, 8-iso-15(R)-PGF_2α_ and 8-iso-13,14-dihydro-PGF_2α_), F- and E-series PGs (PGF_2α_, 13,14-dihydro-PGF_2α_, PGE_1_ and PGE_2_) and two NO_2_-FAs mediators (NO_2_-linoleic acid and NO_2_-oleic acid) (see ESM Table S6).

**Fig. 1:**
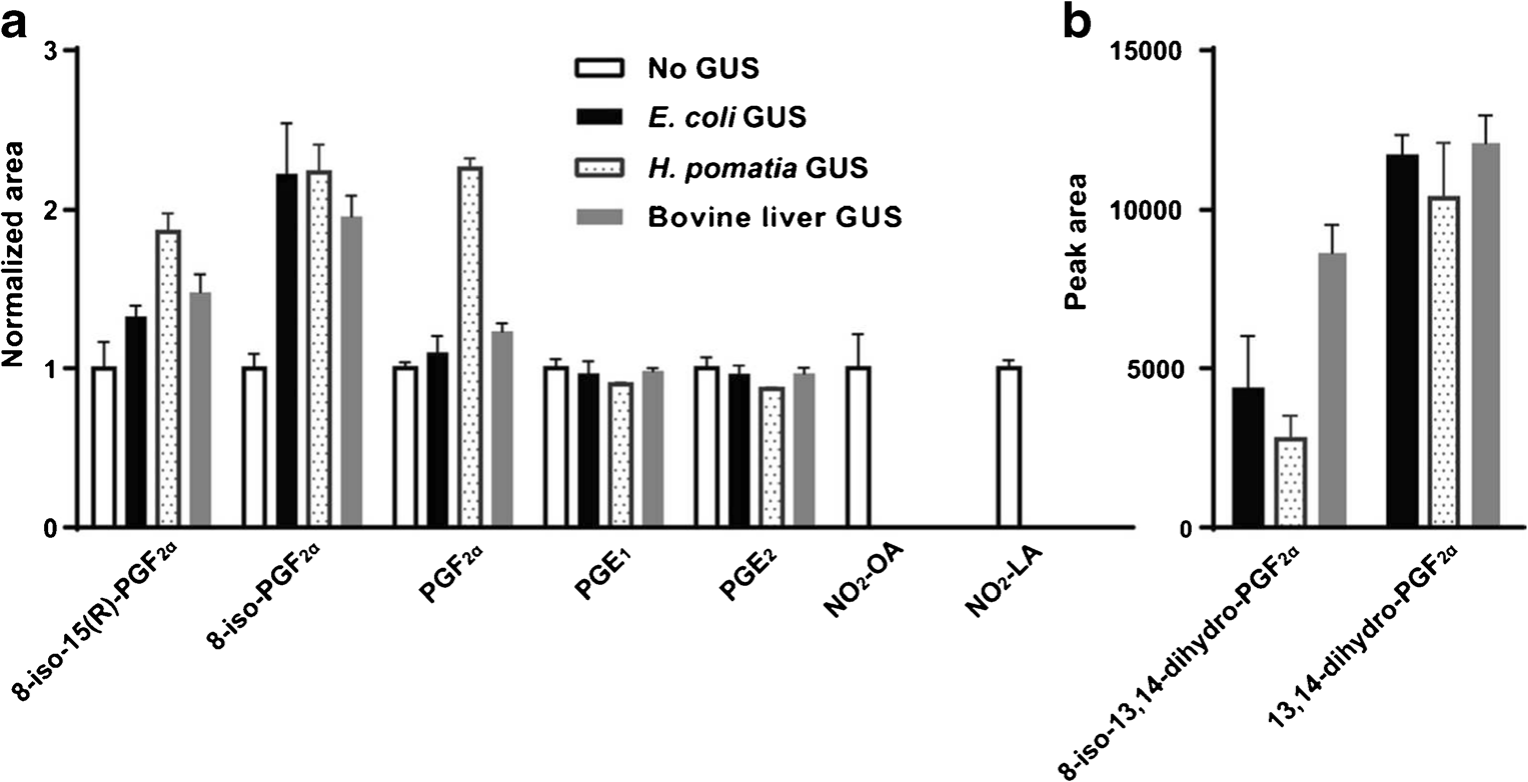
Changes in response of the selected panel of compounds in the urine samples after 2 h enzymatic hydrolysis (at 37 °C with *E. coli*, *H. pomatia* or bovine liver GUS) compared with non-hydrolysed samples (no GUS). **A** *y*-axis represents the normalised peak area of a metabolite normalised to the mean area of the corresponding peak in non-hydrolysed urine. **B** *y*-axis represents the peak area without normalisation since 8-iso-13,14-dihydro-PGF_2α_ and 13,14-dihydro-PGF_2α_ were exclusively detected in GUS-hydrolysed urine. *Error bars* indicate standard deviation

For all three GUSs tested, the optimal conditions was 1000 U enzyme/400 μL urine incubated at 37 °C for 2 h. No increase in metabolite levels were achieved through increasing the hydrolysis time beyond 2 h or from increasing the temperature (data not shown). Choosing the shortest possible hydrolysis time will also increase the throughput of the method. Figure 1 presents the increase (or decrease) of the concentration (as reflected by the changes of the peak area) in the sample after hydrolysis compared with prior to the hydrolysis for a selected panel of compounds. Significant increases were observed for the F-series IsoPs—all three enzymes increased the metabolite levels by more than 50 %. Some downstream metabolites, 8-iso-13,14-dihydro-PGF_2α_ and 13,14-dihydro-PGF_2α_, were exclusively detected after GUS hydrolysis (Fig. 1b). No significant increase in the E-series PGs compared with the free levels were found after GUS hydrolysis. The NO_2_-FAs mediators could not be analysed after hydrolysis due to their extreme temperature and enzymatic lability (see below for further discussion). No significant differences in the hydrolysis efficiency were found between the three GUSs for the pre-selected panel of compounds. Our final choice of the GUS for our protocol was finally made based on encountered blank effects of GUSs and metabolite stability in presence of the GUS (see below).

#### The enzyme blank effect

**Fig. 2:**
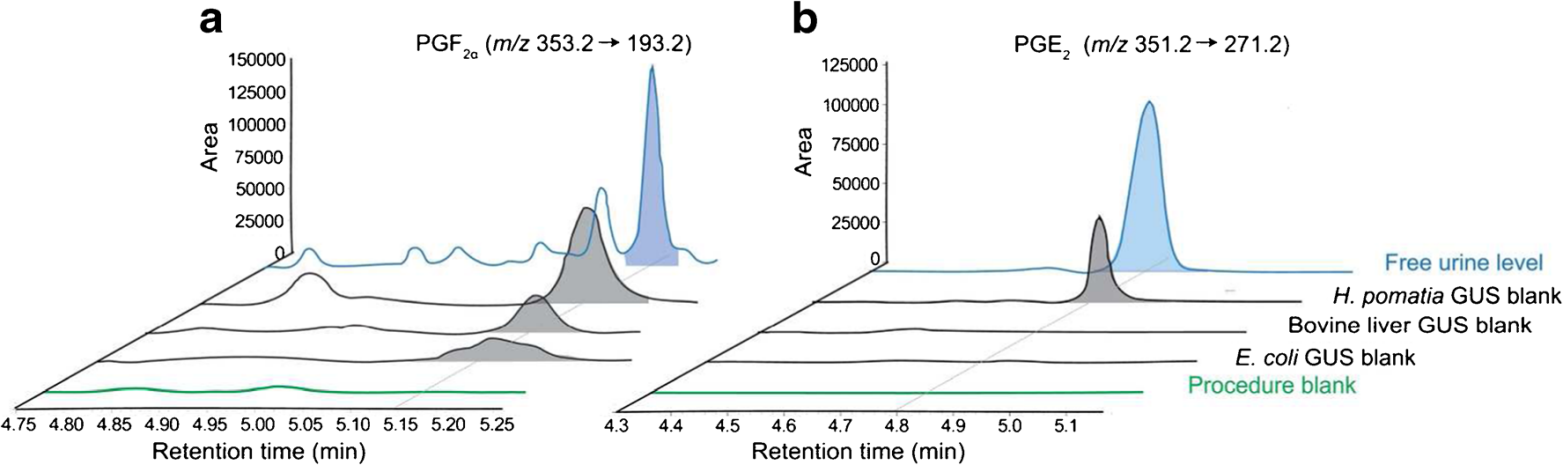
The enzymatic oxidised lipid background (enzyme blank effect). LC-MS/MS chromatograms representing the procedure blank (*green*), followed by the three enzyme blank samples (*E. coli*, bovine liver and *H. pomatia*), and the free urine levels (*blue*) are overlaid for (**A**) PGF_2a_ and (**B**) PGE_2_, respectively. *H.* *pomatia* GUS shows a high oxidised lipid background. Samples were monitored for PGF_2α_ *m/z* 353.2 → 193.2) and PGE_2_ (*m/z* 351.2 → 271.2)

We identified an important and so far unreported observation related to an oxidised lipid background present within the three GUSs, especially for *H. pomatia*. Evaluation of the enzyme blank samples, which consisted of water following the GUS hydrolyses sample workup, revealed the presence of an oxidised lipid background. In Fig. 2, we show the LC-MS/MS trace for PGF_2α_ and PGE_2_ in the non-hydrolysed urine sample, GUS blank samples and procedure blank sample (water sample extracted by LLE, no enzyme added). Figure 2a shows there is no signal for PGF_2α_ in the procedure blank sample while a high level of PGF_2α_ were found especially in the *H. pomatia* GUS blank sample. The levels of PGF_2α_ in *E. coli* and bovine liver GUS samples were lower compared with the high PGF_2α_ background present in *H. pomatia*. Similar observations are made for PGE_2_ (Fig. 2b). For the selected panel of oxidised lipids, the enzyme blank effect is presented by the area ratio between the blank enzyme sample and the hydrolysed sample at 2 h (Area_in enzyme blank sample_/Area_in 2 h hydrolysed sample_). Inspection of the complete IsoP, PG and NO_2_-FA target panel found that *H. pomatia* GUS contained the highest blank effect compared with *E. coli* and bovine liver (see ESM Table S7). Based on this observation, *H. pomatia* was not considered a suitable GUS candidate to measure the total urinary oxidised lipid profile.

#### Internal standard stability

**Fig. 3:**
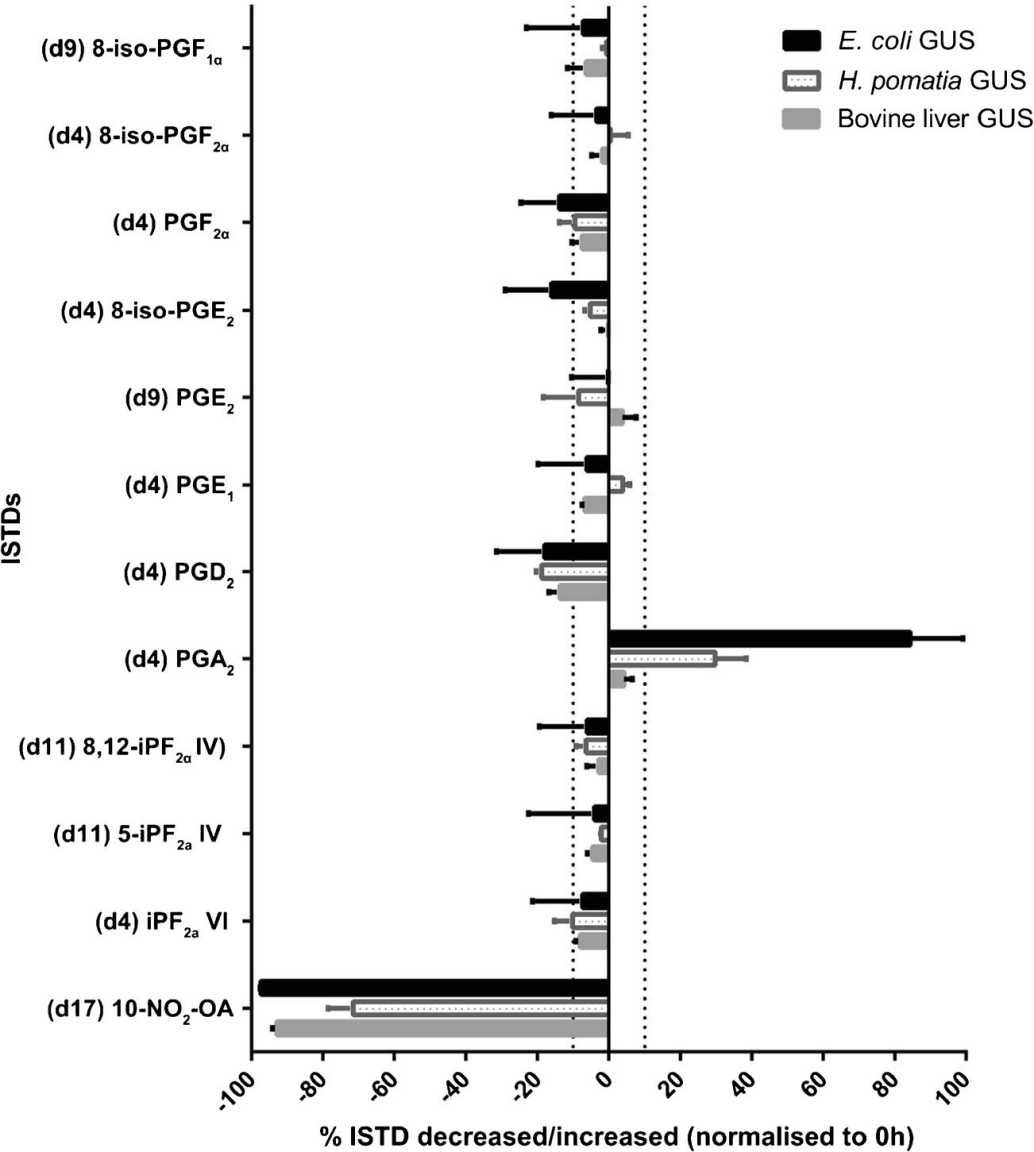
The stability of the IsoP, PG and NO_2_-FA ISTDs during the 2-h hydrolyses. The percentage changes of ISTD levels (compare 2 with 0 h) were investigated to evaluate the stability of each ISTD. Overall, ISTDs with bovine liver GUS hydrolysis indicated the highest degree of stability. The *vertical dotted lines* indicate 10 % change. *x*-axis indicates percentage changes of ISTD areas between 2 and 0 h (area_2 h-ISTD_/area_0 h-ISTD_) × 100 %. *Error bars* indicate standard deviation

Beside the enzyme blank effect, the stability of the ISTDs during the 2 h incubation at 37 °C was investigated as representative for their respective endogenous metabolite classes. The percentage change for the ISTDs treated with the three different enzymes in 2 h compared with 0 h were determined and are shown in Fig. 3. ISTDs representing the F-series PGs and IsoPs were identified as stable at 37 °C, over 2 h with the addition of an enzyme included, showing less than 10 % change in their levels. However, the E- and D-series PG ISTDs showed temperature sensitivity, especially PGD_2_-d4. Reversely, the A-series PG ISTD PGA_2_-d4 showed increasing concentrations, possibly due to PGD_2_-d4 spontaneous dehydration forming PGA_2_-d4. The 10-NO_2_-Oleic acid d17 (NO_2_-FAs ISTD) showed hypersensitivity to hydrolysis conditions (buffer and temperature), showing a 40 % decrease in levels within 2 h compared with non-hydrolysed sample. Furthermore, the addition of all three enzymes resulted in a more pronounced decrease (70 to 90 %), explaining the above-mentioned decrease of endogenous NO2-FAs during enzymatic hydrolyses. Overall, the hydrolysis using GUS from *E. coli* showed the largest percentage change for the evaluated ISTDs, suggesting that the 75 mM phosphate buffer (pH 6.8) or *E. coli* GUS affect compound stability. Although, bovine liver GUS showed similar ISTD stability compared with *H. pomatia*, the latter’s significant enzyme blank effect led to bovine liver being chosen as our preferred hydrolysing enzyme. Furthermore, bovine liver GUS also resulted in the inclusion of D- and A-series PGs in the target list. Therefore, we chose bovine liver-derived GUS hydrolysing at 37 °C for 2 h as the optimal procedure for analysing the urinary oxidised lipid profile.

### Increasing the metabolite scope

Using the optimised bovine liver hydrolyses method, we investigated the potential to broaden the scope of the measurable oxidised urinary lipid profile. We targeted the metabolites from auto-oxidation, COX, LOX and CYP450 pathways and compared the amount of free oxidised lipids (from non-hydrolysed urine) with the total amount of oxidised lipids (from hydrolysed urine). There were 51 metabolites detected in both hydrolysed and non-hydrolysed samples, of which 23 metabolites were significantly increased by hydrolysis. More importantly, 27 additional oxidised lipids were detected exclusively in the total oxidised lipid analysis (Table 1). As shown in Table 1, we were able to increase the scope of the method by using an enzymatic hydrolysis approach to measure the total urinary oxidised lipid signature, providing a more complete picture for biological interpretation.

**Table 1:** Urinary oxidised lipids measured by bovine liver GUS hydrolysis and non-hydrolysis methods

	RNS	ROS	COX	LOX	CYP450
Detected in both GUS-hydrolysed and GUS-non-hydrolysed urine		11-HDoHE*	13,14-dihydro-15-keto-PGE_2_*	12S-HEPE	12,13-DiHOME*
		14-HDoHE	15-keto-PGF_1α_	20-carboxy-LTB_4_^#^	12,13-EpOME*
		5-iPF_2α_- VI*	2,3-dinor-11b-PGF_2α_*	5S,6R-LipoxinA_4_	14,15-DiHETrE*
		8,12-iPF_2α_- IV*	2,3-dinor-8-iso-PGF_2α_*	11-HETE	8,9-DiHETrE*
		8-iso-15(R)-PGF_2α_*	20-hydroxy-PGE_2_^#^	11-trans-LTD4^#^	9,10-DiHOME*
		8-iso-15-keto-PGF_2α_*	Δ12-PGJ_2_*	12-HETE	9,10-EpOME*
		8-iso-PGE_1_^#^	Δ17, 6-ketoPGF_1α_*	13-HODE*	
		8-iso-PGE_2_	PGA_2_	13-KODE	
		8-iso-PGF_1α_	PGD_1_^#^	15-HETE*	
		8-iso-PGF_2α_ *	PGD_2_^#^	5-HETE	
			PGE_1_^#^	9,10,13-TriHOME*	
			PGE_2_	9,12,13-TriHOME*	
			PGE_3_	9-HODE	
			PGF_1α_	9-HOTrE	
			PGF_2α_*	9-KODE	
			PGF_3α_	LTD_4_*	
			PGJ_2_^#^		
			Tetranor-PGEM^#^		
Detected in GUS hydrolysed urine exclusively		10-HDoHE	13_14-dihydro-PGF_2α_	15S-HETrE	20-HETE
		8-iso-13,14-dihydro-PGF_2α_	13,14-dihydro-15-keto-PGD_2_	15-HpETE	12,13-DiHODE
		8-iso-15-keto-PGF_2β_	13,14-dihydro-15-keto-PGF_2α_	5S,15S-DiHETE	19,20-DiHDPA
		9-HETE	15-deoxy-delta-12,14-PGD_2_	5S,6S-Lipoxin A_4_	11,12-DiHETrE
			15-keto-PGF_2α_	5S-HEPE	11,12-EpETrE
			16-HDoHE	5S-HpETE	14,15-DiHETE
			bicyclo-PGE_2_	9-HEPE	17,18-DiHETE
			PGK_2_		5,6-DiHETrE
Detected in non-hydrolysed urine exclusively	NO_2_-αLA				
	NO_2_-LA				
	NO_2_-OA				

*Significantly increased with hydrolysis; ^#^significantly decreased with hydrolysis

### Method validation

Validation measurements were performed using pooled urine as a sample matrix. Recovery, matrix effect and batch-to-batch precision were determined.

#### Recovery and ion suppression

**Fig. 4:**
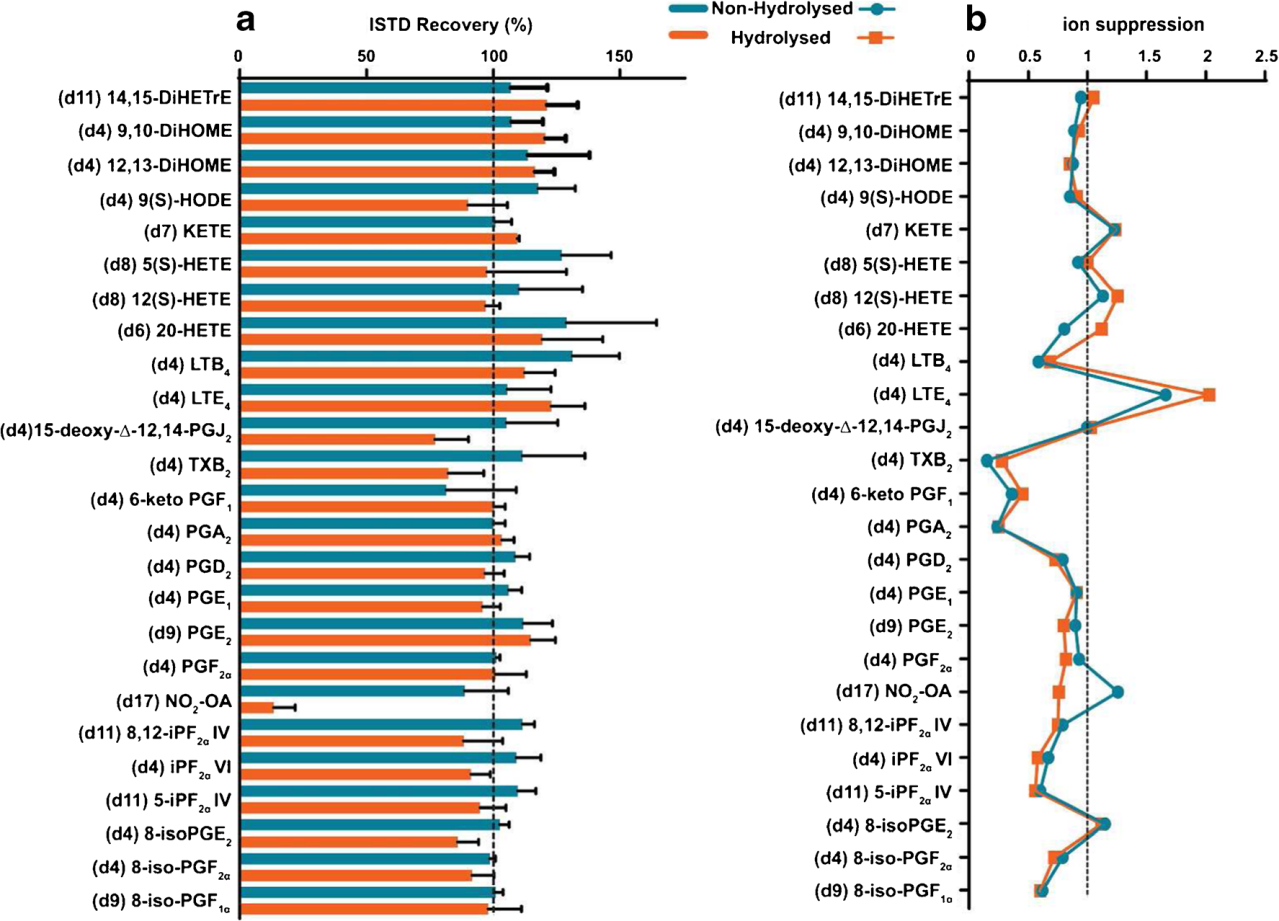
Performance characteristics of sample preparation. Deuterium-labelled ISTDs were evaluated for (**A**) recovery and (**B**) ion suppression; values below 1 indicate presence of ion suppression (**B**)

The performances of the analytical methods (free and total oxidised lipid profiling) with respect to recovery (of the free forms) and ion suppression were validated for those metabolites which were available as deuterium-labelled compounds and which were usually used as ISTDs. For determining recoveries, samples were independently spiked before or spiked after the extraction procedure with ISTDs, and the area ratios between these samples (Area_spike before_/Area_spiked after_) were calculated as the recoveries. Figure 4a demonstrates that recoveries are from 85 to 115 % for most ISTDs, indicating the effectiveness of both procedures to extract the oxidised metabolome from urine samples. The non-hydrolysed samples (free levels) with a simpler sample handling procedure showed slightly higher recoveries compared with the hydrolysed procedure (total levels) except for the nitro-fatty acid ISTD (10-NO_2_-oleic acid-d17 as discussed in “Internal standard stability”).

To determine the ion suppression caused by the presence of compounds of the matrix, ISTD areas in urine samples spiked after the total and free oxidised lipid extractions were compared with the ISTD areas in injection solution. Ion suppression values shown in Fig. 4b ranged between 0.5 and 1 for most of the metabolites; values below 1 indicate the presence of ion suppression caused by co-eluting compounds from the urine matrix that were not removed by the sample preparation and that affect the ionisation of those targeted metabolites [35]. Three ISTDs with high ion suppression (lower than 0.5) were PGA_2_-d4, 6-ketoPGF_1_-d4 and TBX_2_-d4. However, ion suppression effects are corrected through the deuterated ISTD normalisation (Area_metabolite_/Area_ISTD_) as both the metabolite and its corresponding ISTD experience similar ion suppression.

#### Precision and batch-to-batch effect

To be able to reproducibly and accurately report the oxidised lipid metabolome from patient urine samples, it is important to estimate the analytical variation. Therefore, the intra-batch precision and inter-batch variability were assessed for all endogenous oxidised lipids. Precision is dependent on extraction reproducibility, injection variation and detector stability, while the inter-batch variability is influenced by the robustness of the chromatography and instrument stability across different measurement days (*n* = 3).

In ESM Fig. S3a, the precision (intra-batch RSD) and in ESM Fig. S3b, the batch-to-batch effect (inter-batch RSD) of endogenous metabolites obtained from hydrolysed/non-hydrolysed urine samples are shown. Investigating the precision for the non-hydrolysed procedure showed that 59 % of metabolites had the RSD < 15 %, with a further 25 % of metabolites having a RSD between 15 and 30 %. The hydrolysed procedure showed improved precision with 66 % of metabolites having the RSD < 15 % and 29 % of metabolites having a RSD between 15 and 30 %. The increased precision observed in the hydrolysed procedure can be attributed to increased metabolite concentrations, leading to more accurate detection, while the higher variation in the non-hydrolysed procedure was predominantly driven by low abundant compounds. Both procedures performed equally well in batch-to-batch effects, indicative of a stable and reproducible LC-MS analyses across three measurement days.

### Application

#### Subjects

For the present study, baseline urine samples from RA patients who were treated with TNF-α inhibitors (ETN or ADA) were selected based on good response or non-response after 3 months of therapy. The baseline characteristics of 80 patients are shown in Table 2. Subjects had a mean age of 53.8 years, a median disease duration of 5 years and had previously taken a mean of two concomitant disease-modifying anti-rheumatic drugs (co-DMARDs). Subjects were mainly female (58 females, 22 males). Good responders had higher baseline DAS28 (4.8 ± 0.9 compared with 4.2 ± 11, *P* = 0.006) and C-reactive protein (CRP) levels (8 ± 11 compared with 5 ± 7, *P* = 0.04) compared with the non-responders. There were no significant differences between the other parameters.

**Table 2:** Baseline characteristics of the study subjects (*n* = 80), subdivided according to the therapeutic response at 3 months

	All subject (*n* = 80)	Non-response (*n* = 40)	Good response (*n* = 40)	*P* value (good responders vs. non-responders)
Gender (female, *n* (%))	58 (72.5)	31 (77.5)	27 (67.5)	0.453
Age (mean (SD))	53.8 (11.0)	52.7 (11.3)	55.1 (10.8)	0.461
Disease duration (median (IQR))	5 (8.0)	5 (7.0)	6 (9.0)	1.000
Smoking currently (*n* (%))	23 (28.7)	12 (30.0)	11 (27.5)	0.805
Alcohol >7 units/week (*n* (%))	16 (20.3)	5 (12.5)	11 (27.5)	0.099
BMI (mean (SD))	26.9 (5.3)	27.1 (5.0)	26.7 (5.6)	0.874
RF (positive, *n* (%))	57 (71.3)	26 (65.0)	31 (77.5)	0.323
ACPA (positive, *n* (%))	57 (71.3)	26 (65.0)	31 (77.5)	0.323
Baseline DAS28 (mean (SD))	4.5 (1.0)	4.2 (1.1)	4.8 (0.9)	**0.006**
CRP (median (IQR))	7 (10.0)	5 (8)	8 (11.0)	**0.045**

*SD* standard deviation, *IQR* interquartile range

#### Detection of oxidised lipids in human urine

Applying total and free oxidised lipid profiling to randomly collected urine samples, we are able to detect 67 metabolites in hydrolysed urine samples and 44 in non-hydrolysed urine samples. There were 97 % total and 95 % free oxidised lipid metabolites measured with a RSD < 30 % (see ESM Table S8). Because the total oxidised lipid profiling provides a larger scope of metabolites, we focused on the total urinary oxidised lipid profiles (after hydrolysis), in addition to the free NO_2_-FAs levels measured in the analyses without hydrolysis in the subsequent data analyses and interpretation.

#### Relationships between total oxidised lipids profiling and RA-associated parameters

**Fig. 5:**
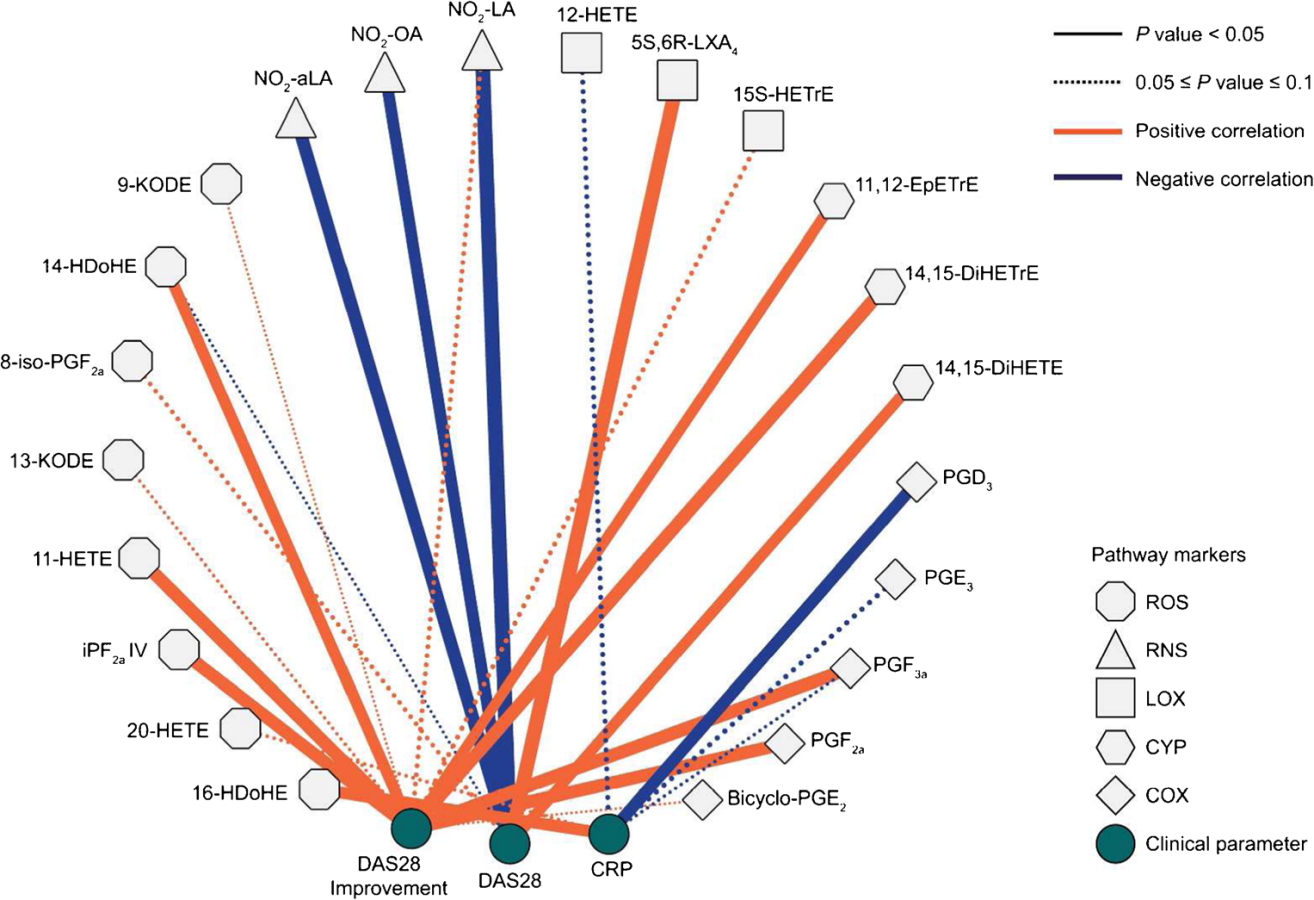
Correlations between rheumatoid arthritis-associated clinical parameters and oxidised lipid levels with *P* < 0.10 based on multiple linear regression analyses

To investigate the relationship between RA-associated parameters and total oxidised lipid levels, repeated assessments using multiple linear regression (MLR) models were performed on individual metabolite. Confounding factors (age, sex, BMI, smoking status, alcohol consumption, co-DMARDs) and RA-associated parameters (baseline DAS28, CRP and DAS28 improvement) were added into MLR as independent variables, and the oxidised lipid level was set as the dependent variable. RA-associated parameters which were correlated with oxidised lipid levels (*P* < 0.10) were selected (Table S9) and are visualised by Cytoscape in Fig. 5. CRP showed a positive correlation with 16-HDoHE (*P* = 0.027) and a negative correlation with PGD_3_ (*P* = 0.026); DAS 28 showed a positive association with 14,15-DiHETE (*P* = 0.036) and 5S, 6R-LipoxinA4 (*P* = 0.005); DAS28 improvement (DAS28_month 0_ − DAS28_month 3_) showed significantly positive association with PGF_3α_ (*P* = 0.017), PGF_2α_ (*P* = 0.018), iPF_2a_ IV (*P* = 0.021), 11,12-EpETrE (*P* = 0.040), 11-HETE (*P* = 0.019), 14,15-DiHETrE (*P* = 0.017) and 14-HDoHE (*P* = 0.033). Since NO_2_-FAs were labile in the hydrolysing procedure, we included the free NO_2_-αLA, NO_2_-LA and NO_2_-OA levels during the MLR. These three metabolites were all negatively associated with baseline DAS28 (*P* < 0.05).

## Discussion

In the present study, we optimised and validated a GUS-based hydrolysis method to evaluate the oxidised urinary lipid profile. We demonstrated that the number of measured oxidised lipids were approximately twofold increased after hydrolysing with bovine liver-sourced GUS. Furthermore, we applied the method to urine samples from patients with RA and identified oxidised lipids associated with RA-associated parameters important in determining therapeutic response.

As a non-invasive biological matrix, urine is easily collected and can effectively be used as an oxidised lipids readout. However, urine does present some challenges including dilution effect (morning sample, 24 h sample, random sample), glucuronidation of metabolites with low solubility, and a high salt concentration etc. Although some of these challenges can be addressed with proper experimental planning and standardised sample collection procedures, the choice between analysing free or total levels of oxidised lipids still needs to be addressed. Thus, we developed a robust enzymatic method to hydrolyse the GlcA-conjugated oxidised lipids, obtaining the total oxidised lipid profile for urine samples. During the method development, we found an enzyme blank effect in the three candidate enzymes (see ESM Table S7) which needs to be minimised in order to avoid adding artefacts which could interfere with the biological interpretation of the results.

Several different GUS are available commercially for research purposes, with each enzyme having its own specific characteristics properties relating to substrate affinities and efficiency. Our choice of candidate enzymes was guided by selecting those previously reported in literature [18, 30, 31]. *H. pomatia* GUS was the most widely used enzyme but had a very prominent blank effect. The oxidised lipid background might be derived from inadequate purifying and cleaning procedures used during enzyme extraction and isolation. While this might not be true for all forms of GUS derived from *H. pomatia*, we decided against the use of *H. pomatia* as the background might influence our biological interpretation of the data.

Comparing the total and free oxidised lipid profiles revealed increased levels, especially in the F-series IsoPs, F-series PGs, hydroxy-fatty acids and dihydroxy-fatty acids, while having minimal effect on E-series PGs in the total oxidised lipid profile. The lack of effect on the E-series PGs could be explained in a study done by Little et al., where they investigated the different human recombinant UDP-glucuronosyltransferases and found that only one isoform UGT2B7 was capable of forming PGE_2_ glucuronide [17]. UGT2B7 were exclusively found in the colon, and faeces rather than urine might contain high levels of E-series glucuronide conjugates. The significant increase in LOX and CYP450 metabolites might be due to their increasing hydrophobic nature, with glucuronidation increasing their urinary excretion. Similar to our findings, Prakash et al., reported significant increase in the level of 20-HETE, an CYP450 metabolite in urine treated with β-glucuronidases, and they concluded that CYP450 metabolites are predominantly excreted in conjugated form [13]. Whereas the method reported by Newman et al. needed 4 mL of urine to measure the free CYP450-oxidised lipid metabolites [22], using our bovine liver hydrolysis method, we could evaluate the same CYP450 pathway metabolites by using ten times less urine.

Optimising the extraction and analyses of the anti-inflammatory NO_2_-FAs will aid us in studying the field of nitrosative stress. A disappointing finding was our inability to accurately measure the total NO_2_-FAs levels, due to their labile nature. Although, we were able to effectively measure the free NO2-αLA, NO_2_-LA and NO_2_-OA levels in urine. In a work published by Salvatore et al., they showed the presence of cysteine-NO_2_-FA conjugates in urine and used a short chemical (HgCl_2_) hydrolysis procedure at 37 °C to measure its total levels [36]. Thus, to accurately measure the total NO_2_-FA level identification of the primary conjugated form needs to be done, followed with optimised chemical hydrolyses.

For the urine samples from RA patients, which were collected at a random time of day, we used the urinary creatinine level for correcting the oxidised lipids for sample dilution. Samples were retrospectively divided into good or no responders to therapy based on EULAR response criteria. However, the subjects could not be separated into non-/good response groups based on their baseline urinary oxidised lipids profile and clinical parameters using categorical principle components analysis (CATPCA, score plot is shown in ESM Fig. S4). After correcting for confounding factors (age, sex, BMI, smoking status, alcohol consumption, co-DMARDs), the urinary oxidised lipid readout did show associations between inflammation and oxidative stress and clinical parameters (CRP, 3-month DAS28 improvement, baseline DAS28). C-reactive protein is an acute-phase inflammatory protein, which showed a positive correlation with 16-HDoHE, a docosahexaenoic acid (DHA) lipid peroxidation metabolite. DHA showed efficacy as an anti-inflammatory treatment when used as a prophylactic treatment in a mouse RA model [37], thus the increased peroxidation of DHA catalysed by ROS reduces the body’s anti-inflammatory capacity. Oxidative stress has been identified as an aggravator of damage caused to bone in cartilage in RA [38, 39], so its correlation with CRP underscores this relationship.

DAS28 is the most widely used measurement for assessing the disease activity (swelling and tenderness in 28 joints, the ESR and VAS general health) in RA [40]. In the present study, good responders had higher baseline DAS28 compared with non-responders. Clinically, it has been observed that a patient with severe RA has high DAS28 and is more likely to obtain therapeutic response [41]. Veselinovic et al. reported increased levels of RNS in patients with high disease activity in serum [38], while we found in urine that the downstream anti-inflammatory NO_2_-FAs correlated negatively with DAS28. This observation could be explained through the complex relationship between RNS species, the beneficial signalling abilities of RNS-nitrated lipid metabolites (NO_2_-FAs) and the body’s anti-oxidant capacity. The positive correlation of DAS28 improvement with strong pro-inflammatory mediators PGF_2α_, PGF_3α_ and oxidative stress markers iPF_2α_-IV, 11-HETE and 14-HDoHE indicates the higher disease burden within these baseline patients. The correlation of DAS28 and its improvement with the anti-inflammatory CYP-450 dihydroxy-fatty acids metabolites and the LOX derived LXA_4_ possibly reflects the intact innate anti-inflammatory pathways in these patients still trying to lessen the RA disease burden. The dihydroxy-fatty acids, 14,15-DiHETE, 11,12-EpETrE and 14,15-DiHETrE are able to attenuate pro-inflammatory pathways through activating and signalling via the PPAR-gamma pathway [42]. The urinary oxidised lipid profile of RA patients indicates the complex nature of the disease with oxidative stress and inflammation having an intimate relationship with clinically measured parameters.

## Conclusions

In the present study, we thoroughly explored different deglucoronidation methods for urinary oxidised lipids and developed a bovine liver GUS hydrolysing sample preparation method coupled with LC-MS to analyse the total urinary oxidised lipid profile. With bovine liver GUS hydrolysis, we are able to zoom into the urinary oxidised lipid metabolome, providing a readout for inflammation and oxidative stress. Our method detected more than 70 oxidised lipids in urine samples, biosynthesised from two non-enzymatic and three enzymatic pathways. The total oxidised lipid profiling method was developed and validated for human urine and was demonstrated on patients with RA. The urinary oxidised lipid profile of RA patients indicates the complex nature of the disease with oxidative stress and inflammation having an intimate relationship with clinically measured parameters. In conclusion, the hydrolysed method developed here allows specific and sensitive quantitative assessment of more than 70 oxidised lipids, which expands the scope of compounds in urinary metabolic profiling and may have wider applications in studies elucidating the role of these potent bioactive metabolites in human diseases.

## Electronic supplementary material

Below is the link to the electronic supplementary material.

ESM 1 (PDF 1.01 mb)
