# Supplementary material for: Metabolomics profiling of the free and total oxidised lipids in urine by LC-MS/MS: application in patients with rheumatoid arthritis
Source: Anal Bioanal Chem. 2016 Jul 12;408(23):6307–19. doi: 10.1007/s00216-016-9742-2 (PMC5009176; doi:10.1007/s00216-016-9742-2)
Supplement: Supplementary file 1 — (PDF 1.01 mb) [file 216_2016_9742_MOESM1_ESM.pdf]

Analytical and Bioanalytical Chemistry

Electronic Supplementary Material

Metabolomics profiling of the free and total oxidised lipids in urine by LC-MS/MS: application in patients with rheumatoid arthritis

Junzeng Fu, Johannes C. Schoeman, Amy C. Harms, Herman A. van Wietmarschen, Rob J. Vreeken, Ruud Berger, Bart V.J. Cuppen, Floris P.J.G. Lafeber, Jan van der Greef, Thomas Hankemeier

Tables

Table S1 Overview of internal standards

ISTD name	*m/z* Precursor ion -> Product ion
(±) 12,13-DiHOME-d4	317.3 -> 185.2
(±) 9,10-DiHOME-d4	317.3 -> 203.2
10-Nitrooleate-d17	343.1->46.05
12(S)-HETE-d8	327.3 -> 184.2
14,15-DiHETrE-d11	348.3 -> 207.1
15-deoxy-D-13,14-PGJ2-d4	319.2 -> 275.3
20-HETE-d6	325.3 -> 279.2
5(S)-HETE-d8	327.3 -> 116.1
5-iPF2a-VI-d11	364.2->115.05
6-keto PGF1a-d4	373.2 -> 167.2
8,12-iso-iPF2a-d11	364.2->115.05
8-iso-PGE2-d4	355.3->275.25
8-iso-PGF1a-d9	364.2->320.25
8-iso-PGF2a-d4	357.3->197.15
9(S)-HODE-d4	299.2 -> 172.1
iPF2a-VI-d4	357.2->114.9
KETE-d7	324.2 -> 280.3
LTB4-d4	339.2 -> 197.1
LTE4-d3	441.2 -> 336.2
PGA2-d4	336.9->275.0
PGD2-d4	355.2 -> 275.2
PGE1-d4	357.3->321.2
PGE2-d9	359.9->280.25
PGE2-d9	359.9->280.25
PGF2a-d4	357.3-> 197.2
TXB2-d4	373.2-> 173.1

Table S2 Complete COX pathway oxidised lipid target list

Metabolites	Precursor ion Product ion (*m/z*)	ISTD	Formal name	Lipidmaps ID	InChlKey
11beta-13,14-dihydro-15-keto-PGF2a	353.2 -> 113.2	(d4) PGF2a	9?,11?-dihydroxy-15-oxo-prost-5Z-en-1-oic acid	LMFA03010203	VKTIONYPMSCHQI-KGILNJEGSA-N
11beta-PGE2	351.2 -> 271.3	(d9) PGE2	9-oxo-11?,15S-dihydroxy-prosta-5Z,13E-dien-1-oic acid	LMFA03010060	XEYBRNLFEZDVAW-YUOXZBOXSA-N
11beta-PGF2a	353.2 -> 193.1	(d4) PGF2a	9α,11β,15S-trihydroxy-prosta-5Z,13E-dien-1-oic acid	LMFA03010036	PXGPLTODNUVGFL-ZWAKLXPCSA-N
12S-HHTrE	279.2 -> 179.2	(d8) 12(S)-HETE	12S-hydroxy-5Z,8E,10E-heptadecatrienoic acid	LMFA03050002	KUKJHGXXZWHSBG-WBGSEQOASA-N
13,14-dihydro-15-keto-PGD1	353.2 -> 209.1	(d4) PGD2	9α-hydroxy-11,15-dioxo-prost-1-oic acid	*na*	WTCAXDJXNVRHRC-KURKYZTESA-N
13,14-dihydro-15-keto-PGD2	351.2 -> 175.2	(d4) PGD2	9?-hydroxy-11,15-dioxo-prost-5Z-en-1-oic acid	LMFA03010022	VSRXYLYXIXYEST-KZTWKYQFSA-N
13,14-dihydro-15-keto-PGE2	351.2 -> 175.2	(d9) PGE2	9,15-dioxo-11?-hydroxy-prost-5Z-en-1-oic acid	LMFA03010031	CUJMXIQZWPZMNQ-XYYGWQPLSA-N
13,14-dihydro-15-keto-PGF1a	355.2 -> 193.2	(d4) PGF2a	9S,11R-dihydroxy-15-oxo-prostanoic acid	LMFA03010168	FVPKMMQYALWZHV-AKHDSKFASA-N
13,14-dihydro-15-keto-PGF2a	353.2 -> 183.1	(d4) PGF2a	9?,11?-dihydroxy-15-oxo-prost-5Z-en-1-oic acid	LMFA03010027	VKTIONYPMSCHQI-XAGFEHLVSA-N
13,14-dihydro-PGF2a	355.2 -> 275.3	(d4) PGF2a	9S,11R,15S-trihydroxy-5Z-prostenoic acid	LMFA03010079	LLQBSJQTCKVWTD-QPJJXVBHSA-N
15-deoxy-delta-12,14-PGD2	333.2 -> 271.2	(d4) PGD2	9?-hydroxy-11-oxo-prosta-5Z,12E,14E-trien-1-oic acid	LMFA03010051	QUGBPWLPAUHDTI-PLGLXCLHSA-N
15-keto-PGF1a	353.2 -> 221.1	(d4) PGF2a	9S,11R-dihydroxy-15-oxo-13E-prostaenoic acid	LMFA03010150	QPXXPLNAYDQELM-QNXXGYPUSA-N
15-keto-PGF2a	351.2 -> 219.1	(d4) PGF2a	9S,11R-dihydroxy-15-oxo-5Z,13E-prostadienoic acid	LMFA03010026	LOLJEILMPWPILA-AMFHKTBMSA-N
1a,1b-dihomo-PGF2a	381.3 -> 337.2	(d4) PGF2a	1a,1b-dihomo-9S,11R,15S-trihydroxy-5Z,13E-prostadienoic acid	LMFA03010157	ZCTAOAWRUXSOQF-GWSKAPOCSA-N
2,3-dinor-11b-PGF2a	325.2 -> 145.1	(d4) 8-iso-PGF2a	9α,11β,15S-trihydroxy-2,3-dinor-prosta-5Z,13E-dien-1-oic acid	LMFA03010011	IDKLJIUIJUVJNR-KSJYGFEGSA-N
20-hydroxy-PGE2	367.2 -> 287.2	(d9) PGE2	9-oxo-11?,15S,20-trihydroxy-prosta-5Z,13E-dien-1-oic acid	LMFA03010014	AZIGEYVZEVXWAD-NZGURKHLSA-N
20-hydroxy-PGF2a	369.2 -> 193.1	(d4) PGF2a	9S,11S,15S,20-tetrahydroxy-5Z,13E-prostadienoic acid	LMFA03010029	XQXUYZDBZCLAQO-UNKHNRNISA-N
6-keto PGE1	367.2 -> 143.1	(d4) 6-keto PGF1a	6,9-dioxo-11R,15S-dihydroxy-13E-prostenoic acid	LMFA03010012	ROUDCKODIMKLNO-CTBSXBMHSA-N
6-keto-PGF1a	369.2 -> 163.1	(d4) 6-keto PGF1a	6-oxo-9?,11?,15S-trihydroxy-prost-13E-en-1-oic acid	LMFA03010001	KFGOFTHODYBSGM-ZUNNJUQCSA-N
bicyclo-PGE2	333.2 -> 113.2	(d9) PGE2	11-deoxy-13,14-dihydro-15-keto-11?,16.xi.-cycloprostaglandin E2	LMFA03010034	CGCZPIJMGKLVTQ-PAJBVNRRSA-N
d12-PGJ2	333.2 -> 233.1	(d4) 15-deoxy-D-13,14-PGJ2	11-oxo-15S-hydroxy-prosta-5Z,9,12E-trien-1-oic acid	LMFA03010020	TUXFWOHFPFBNEJ-GJGHEGAFSA-N
D17, 6-ketoPGF1a	367.2 -> 163.1	(d4) 6-keto PGF1a	6-oxo-9S,11R,15S-trihydroxy-13E,17Z-prostadienoic acid	LMFA03010149	HFKNJQYMAGMXTR-CAPHXMBKSA-N

PGA1	335.1 -> 273.15	(d4) PGA2	9-oxo-15S-hydroxy-10Z,13E-prostadienoic acid	LMFA03010005	BGKHCLZFGPIKKU-LDDQNKHRSA-N
PGA2	333.2 -> 271.2	(d4) PGA2	9-oxo-15S-hydroxy-5Z,10Z,13E-prostatrienoic acid	LMFA03010035	MYHXHCUNDDAEOZ-FOSBLDSVSA-N
PGD1	353.2 -> 273.2	(d4) PGD2	9?,15S-dihydroxy-11-oxo-prost-13E-en-1-oic acid	LMFA03010049	CIMMACURCPXICP-PNQRDDRVSA-N
PGD2	351.2 -> 271.2	(d4) PGD2	9S,15S-dihydroxy-11-oxo-5Z,13E-prostadienoic acid	LMFA03010004	BHMBVRSPMRCCGG-OUTUXVNYSA-N
PGD3	349.2 -> 269.2	(d4) PGD2	9S,15S-dihydroxy-11-oxo-5Z,13E,17Z-prostatrienoic acid	LMFA03010142	ANOICLBSJIMQTA-WXGBOJPQSA-N
PGE1	353.2 -> 273.2	(d4) PGE1	9-oxo-11R,15S-dihydroxy-13E-prostaenoic acid	LMFA03010134	GMVPRGQOIOIIMI-DWKJAMRDSA-N
PGE2	351.2 -> 271.2	(d9) PGE2	9-oxo-11R,15S-dihydroxy-5Z,13E-prostadienoic acid; Prostin E2	LMFA03010003	XEYBRNLFEZDVAW-ARSRFYASSA-N
PGE3	349.2 -> 269.2	(d9) PGE2	9-oxo-11R,15S-dihydroxy-5Z,13E,17Z-prostatrienoic acid	LMFA03010135	CBOMORHDRONZRN-QLOYDKTKSA-N
PGF1a	355.2 -> 293.2	(d4) PGF2a	9S,11R,15S-trihydroxy-13E-prostaenoic acid	LMFA03010137	DZUXGQBLFALXCR-CDIPTNKSSA-N
PGF2a	353.2 -> 193.1	(d4) PGF2a	9S,11R,15S-trihydroxy-5Z,13E-prostadienoic acid	LMFA03010002	PXGPLTODNUVGFL-YNNPMVKQSA-N
PGF3a	351.2 -> 193.2	(d4) PGF2a	9α,11α,15S-trihydroxy-prosta-5Z,13E,17Z-trien-1-oic acid	LMFA03010138	SAKGBZWJAIABSY-SAMSIYEGSA-N
PGJ2	333.2 -> 233.1	(d4) PGD2	11-oxo-15S-hydroxy-prosta-5Z,9,13E-trien-1-oic acid	LMFA03010019	UQOQENZZLBSFKO-POPPZSFYSA-N
PGK2	349.2 -> 205.1	(d9) PGE2	9,11-dioxo-15S-hydroxy-5Z,13E-prostadienoic acid	LMFA03010023	LGMXPVXJSFPPTQ-DJUJBXLVSA-N
Tetranor-PGEM	327.1 -> 309.2	(d9) PGE2	11R-hydroxy-9,15-dioxo-2,3,4,5-tetranor-prostan-1,20-dioic acid	LMFA03010032	ZJAZCYLYLVCSNH-JHJVBQTASA-N
Tetranor-PGFM	329.2 -> 311.2	(d4) PGF2a	9S,11R-dihydroxy-15-oxo-2,3,4,5-tetranor-prostan-1,20-dioic acid	LMFA03010139	IGRHJCFWWOQYQE-SYQHCUMBSA-N
TXB1	371.2 -> 171.1	(d4) TXB2	9S,11,15S-trihydroxy-thrombox-13E-enoic acid	LMFA03030008	JSDWWNLTJCCSAV-RLBQWBRQSA-N
TXB2	369.2 -> 169.1	(d4) TXB2	9S,11,15S-trihydroxy-thromboxa-5Z,13E-dien-1-oic acid	LMFA03030002	XNRNNGPBEPRNAR-JQBLCGNGSA-N
TXB3	367.2 -> 169.1	(d4) TXB2	9S,11,15S-trihydroxy-thromboxa-5Z,13E,17Z-trien-1-oic acid	LMFA03030006	OYPPJMLKAYYWHH-NXJDUNGTSA-N

Table S3 Complete CYP pathway oxidised lipid target list

Metabolites	Precursor ion □ Product ion (*m/z*)	ISTD	Formal name	Lipidmaps ID	InChlKey
11,12-DiHETrE	337.2 -> 167.2	(d11) 14,15-DiHETrE	(±)11,12-dihydroxy-5Z,8Z,14Z-eicosatrienoic acid	LMFA03050008	LRPPQRCHCPFBPE-LZXKBWHHSA-N
11,12-EpETrE	319.2 -> 167.1	(d11) 14,15-DiHETrE	(±)11(12)-epoxy-5Z,8Z,14Z-eicosatrienoic acid	LMFA03080014	DXOYQVHGIODESM-LZXKBWHHSA-N
12,13-DiHODE	311.2 -> 293.0	(d4) 9(S)-HODE	(+/-)-12,13-dihydroxy-9Z,15Z-octadecadienoic acid	LMFA02000046	RGRKFKRAFZJQMS-OOHFSOINSA-N
12,13-DiHOME	313.2 -> 183.2	(d4) 12,13-DiHOME	12,13-dihydroxy-9Z-octadecenoic acid	LMFA01050351	CQSLTKIXAJTQGA-GJGKEFFFSA-N
12,13-EpOME	295.2 -> 195.2	(d4) 12,13-DiHOME	(+/-)-12(13)-epoxy-9Z-octadecenoic acid	LMFA02000038	CCPPLLJZDQAOHD-FLIBITNWSA-N
14,15-DiHETE	335.2 -> 207.1	(d11) 14,15-DiHETrE	14,15-dihydroxy-5Z,8Z,11Z,17Z-eicosatetraenoic acid	LMFA03060077	BLWCDFIELVFRJY-IXQKDQKQSA-N
14,15-DiHETrE	337.2 -> 207.2	(d11) 14,15-DiHETrE	(±)14,15-dihydroxy-5Z,8Z,11Z-eicosatrienoic acid	LMFA03050010	SYAWGTIVOGUZMM-KZTFMOQPSA-N
14,15-EpETE	317.2 -> 207.1	(d11) 14,15-DiHETrE	(±)14(15)-epoxy-5Z,8Z,11Z,17Z-eicosatetraenoic acid	LMFA03000003	RGZIXZYRGZWDMI-IXQKDQKQSA-N
14,15-EpETrE	319.2 -> 219.2	(d11) 14,15-DiHETrE	(±)14(15)-epoxy-5Z,8Z,11Z-eicosatrienoic acid	LMFA03080013	JBSCUHKPLGKXKH-KZTFMOQPSA-N
16,17-EpDPE	343.2 -> 233.2	(d11) 14,15-DiHETrE	(±)16(17)-epoxy-4Z,7Z,10Z,13Z,19Z-docosapentaenoic acid	LMFA04000037	BCTXZWCPBLWCRV-QCAYAECISA-N
17,18-DiHETE	335.2 -> 247.2	(d11) 14,15-DiHETrE	(+/-)-17,18-dihydroxy-5Z,8Z,11Z,14Z-eicosatetraenoic acid	LMFA03060078	XYDVGNAQQFWZEF-JPURVOHMSA-N
17,18-EpETE	317.2 -> 259.2	(d11) 14,15-DiHETrE	(+/-)-17(18)-epoxy-5Z,8Z,11Z,14Z-eicosatetraenoic acid	LMFA03000004	GPQVVJQEBXAKBJ-JPURVOHMSA-N
19,20-DiHDPA	361.2 -> 273.3	(d11) 14,15-DiHETrE	(±)19,20-dihydroxy-4Z,7Z,10Z,13Z,16Z-docosapentaenoic acid	LMFA04000043	FFXKPSNQCPNORO-MBYQGORISA-N
20-HETE	319.2 -> 289.2	(d6) 20-HETE	20-hydroxy-5Z,8Z,11Z,14Z-eicosatetraenoic acid	LMFA03060009	NNDIXBJHNLFJJP-DTLRTWKJSA-N
5,6-DiHETrE	337.2 -> 145.1	(d11) 14,15-DiHETrE	5S,6S-dihydroxy-7E,9E,11Z,14Z-eicosatetraenoic acid	LMFA03060018	UVZBUUTTYHTDRR-WAQVJNLQSA-N
5,6-EpETrE	319.2 -> 191.2	(d11) 14,15-DiHETrE	(±)5(6)-epoxy-8Z,11Z,14Z-eicosatrienoic acid	LMFA03080017	VBQNSZQZRAGRIX-GSKBNKFLSA-N
5S,6S-DiHETE	335.2 -> 115.1	(d4) LTB4	5S,6S-dihydroxy-7E,9E,11Z,14Z-eicosatetraenoic acid	LMFA03060018	UVZBUUTTYHTDRR-WAQVJNLQSA-N
8,9-DiHETrE	337.2 -> 127.0	(d11) 14,15-DiHETrE	8,9-dihydroxy-5Z,11Z,14Z-eicosatrienoic acid	LMFA03050006	DCJBINATHQHPKO-TYAUOURKSA-N
8,9-EpETrE	319.2 -> 155.1	(d11) 14,15-DiHETrE	(±)8(9)-epoxy-5Z,11Z,14Z-eicosatrienoic acid	LMFA03080019	DBWQSCSXHFNTMO-ZZMPYBMWSA-N
9,10-DiHOME	313.2 -> 201.1	(d4) 9,10-DiHOME	9,10-dihydroxy-12Z-octadecenoic acid	LMFA02000229	XEBKSQSGNGRGDW-CJWPDFJNSA-N
9,10-EpOME	295.2 -> 171.2	(d4) 9,10-DiHOME	(+/-)-9(10)-epoxy-12Z-octadecenoic acid	LMFA02000037	FBUKMFOXMZRGRB-XKJZPFPASA-N

Table S4 Complete LOX pathway oxidised lipid target list

Metabolites	Precursor ion Product ion (*m/z*)	ISTD	Formal name	Lipidmaps ID	InChlKey
10S,17S-DiHDoHE	359.2 -> 153.2	(d4) LTB4	10(S),17(S)-dihydroxy-4Z,7Z,11E,13Z,15E,19Z-docosahexaenoic acid	LMFA04000047	CRDZYJSQHCXHEG-XLBFCUQGSA-N
11-trans-LTC4	624.3 -> 272.1	(d4) LTE4	5S-hydroxy-6R-(S-glutathionyl)-7E,9E,11E,14Z-eicosatetraenoic acid	LMFA03020020	GWNVDXQDILPJIG-GXMXCQGXSA-N
11-trans-LTD4	495.2 -> 177.1	(d4) LTE4	5S-hydroxy-6R-(S-cysteinylglycinyl)-7E,9E,11E,14Z-eicosatetraenoic acid	LMFA03020021	YEESKJGWJFYOOK-XILINCIBSA-N
11-trans-LTE4	438.2 -> 333.2	(d4) LTE4	5S-hydroxy-6R-(S-cysteinyl)-7E,9E,11E14Z-eicosatetraenoic acid	LMFA03020022	OTZRAYGBFWZKMX-DVFCZEDWSA-N
12-HETE	319.2 -> 179.2	(d8) 12(S)-HETE	12-hydroxy-5Z,8Z,10E,14Z-eicosatetraenoic acid	LMFA03060088	ZNHVWPKMFKADKW-VXBMJZGYSA-N
12-KETE	317.2 -> 273.3	(d7) KETE	12-oxo-5Z,8Z,10E,14Z-eicosatetraenoic acid	LMFA03060019	GURBRQGDZZKITB-VXBMJZGYSA-N
12S-HEPE	317.2 -> 179.1	(d8) 12(S)-HETE	12S-hydroxy-5Z,8Z,10E,14Z,17Z-eicosapentaenoic acid	LMFA03070008	MCRJLMXYVFDXLS-UOLHMMFFSA-N
12S-HpETE	317.2 -> 273.3	(d8) 12(S)-HETE	12S-hydroperoxy-5Z,8Z,10E,14Z-eicosatetraenoic acid	LMFA03060013	ZIOZYRSDNLNNNJ-VXBMJZGYSA-N
13-HODE	295.2 -> 195.2	(d4) 9(S)-HODE	13S-hydroxy-9Z,11E-octadecadienoic acid	LMFA02000154	HNICUWMFWZBIFP-BSZOFBHHSA-N
13-HpODE	311.2 -> 113.2	(d4) 9(S)-HODE	(±)13-hydroperoxy-9Z,11E-octadecadienoic acid	LMFA02000034	JDSRHVWSAMTSSN-BSZOFBHHSA-N
13-KODE	293.2 -> 113.1	(d4) 9(S)-HODE	13-keto-9Z,11E-octadecadienoic acid	LMFA02000016	JHXAZBBVQSRKJR-BSZOFBHHSA-N
14,15-LTC4	624.3 -> 272.1	(d4) LTE4	15S-hydroxy-14R-(S-glutathionyl)-5Z,8Z,10E,12E-eicosatetraenoic acid	LMFA03020031	OBQVBASHEWLKCQ-JTLMKIFUSA-N
14,15-LTE4	438.2 -> 333.2	(d4) LTE4	15S-hydroxy,14R-(S-cysteinyl)-5Z,8Z,10E,12E-eicosatetraenoic acid	LMFA03020033	JLJNENVYAVKECZ-HRXVJLLUSA-N
15-HETE	319.2 -> 219.2	(d8) 5(S)-HETE	15S-hydroxy-5Z,8Z,11Z,13E-eicosatetraenoic acid	LMFA03060001	JSFATNQSLKRBCI-VAEKSGALSA-N
15-HpETE	335.2 -> 113.1	(d8) 5(S)-HETE	15S-hydroperoxy-5Z,8Z,11Z,13E-eicosatetraenoic acid	LMFA03060014	BFWYTORDSFIVKP-VAEKSGALSA-N
15-KETE	317.2 -> 113.2	(d7) KETE	15-oxo-5Z,8Z,11Z,13E-eicosatetraenoic acid	LMFA03060051	YGJTUEISKATQSM-USWFWKISSA-N
15S-HEPE	317.2 -> 219.2	(d8) 5(S)-HETE	15S-hydroxy-5Z,8Z,11Z,13E,17Z-eicosapentaenoic acid	LMFA03070009	WLKCSMCLEKGITB-DBVSHIMFSA-N
15S-HETrE	321.2 -> 221.2	(d8) 5(S)-HETE	15S-hydroxy-8Z,11Z,13E-eicosatrienoic acid	LMFA03050007	IUKXMNDGTWTNTP-OAHXIXLCSA-N
20-carboxy-LTB4	365.2 -> 347.2	(d4) LTB4	5S,12R-dihydroxy-6Z,8E,10E,14Z-eicosatetraene-1,20-dioic acid	LMFA03020016	SXWGPVJGNOLNHT-VFLUTPEKSA-N
20-hydroxy-LTB4	351.2 -> 195.1	(d4) LTB4	5S,12R,20-trihydroxy-6Z,8E,10E,14Z-eicosatetraenoic acid	LMFA03020018	PTJFJXLGRSTECQ-PSPARDEHSA-N
5-HETE	319.2 -> 115.1	(d8) 5(S)-HETE	5S-hydroxy-6E,8Z,11Z,14Z-eicosatetraenoic acid	LMFA03060002	KGIJOOYOSFUGPC-JGKLHWIESA-N
5-HETrE	321.3 -> 303.0	(d8) 5(S)-HETE	5S-hydroxy-6E,8Z,11Z-eicosatrienoic acid	LMFA03050005	LSADDRSUZRRBAN-FDSUASFTSA-N

5-KETE	317.2 -> 203.2	(d7) KETE	5-oxo-6E,8Z,11Z,14Z-eicosatetraenoic acid	LMFA03060011	MEASLHGILYBXFO-XTDASVJISA-N
5S,14R-Lipoxin B4	351.2 -> 221.2	(d4) LTB4	5S,14R,15S-trihydroxy-6E,8Z,10E,12E-eicosatetraenoic acid	LMFA03040002	UXVRTOKOJOMENI-WLPVFMORSA-N
5S,15S-DiHETE	335.2 -> 115.1	(d4) LTB4	5S,15S-dihydroxy-6E,8Z,10Z,13E-eicosatetraenoic acid	LMFA03060010	UXGXCGPWGSUMNI-BVHTXILBSA-N
5S,6R-LipoxinA4	351.2 -> 115.1	(d4) LTB4	5S,6R,15S-trihydroxy-7E,9E,11Z,13E-eicosatetraenoic acid	LMFA03040001	IXAQOQZEOGMIQS-SSQFXEBMSA-N
5S,6S-Lipoxin A4	351.2 -> 115.1	(d4) LTB4	5S,6R,15S-trihydroxy-7E,9E,11Z,13E-eicosatetraenoic acid	LMFA03040001	IXAQOQZEOGMIQS-SSQFXEBMSA-N
5S-HEPE	317.2 -> 115.1	(d8) 5(S)-HETE	5S-hydroxy-6E,8Z,11Z,14Z,17Z-eicosapentaenoic acid	LMFA03070010	FTAGQROYQYQRHF-GHWNLOBHSA-N
5S-HpETE	317.3 -> 203.2	(d8) 5(S)-HETE	5S-hydroperoxy-6E,8Z,11Z,14Z-eicosatetraenoic acid	LMFA03060012	JNUUNUQHXIOFDA-JGKLHWIESA-N
6-trans-LTB4	335.2 -> 195.2	(d4) LTB4	5S,12R-dihydroxy-6E,8E,10E,14Z-eicosatetraenoic acid	LMFA03020013	VNYSSYRCGWBHLG-UKNWISKWSA-N
8-HETrE	321.3 -> 303.0	(d8) 12(S)-HETE	8S-hydroxy-9E,11Z,14Z-eicosatrienoic acid	LMFA03050011	SKIQVURLERJJCK-RDCCVJQZSA-N
8S,15S-DiHETE	335.2 -> 235.2	(d4) LTB4	8S,15S-dihydroxy-5Z,9E,11Z,13E-eicosatetraenoic acid	LMFA03060050	NNPWRKSGORGTIM-HCCKYKKOSA-N
9,10,13-TriHOME	329.2 -> 171.1	(d4) 9(S)-HODE	E)-9,10,13-Trihydroxy-11-octadecenoic acid	LMFA02000168	NTVFQBIHLSPEGQ-BUHFOSPRSA-N
9,12,13-TriHOME	329.2 -> 211.2	(d4) 9(S)-HODE	9S,12S,13S-trihydroxy-10E-octadecenoic acid	LMFA02000014	MDIUMSLCYIJBQC-MVFSOIOZSA-N
9-HODE	295.2 -> 171.1	(d4) 9(S)-HODE	(±)-9-hydroxy-10E,12Z-octadecadienoic acid	LMFA02000151	NPDSHTNEKLQQIJ-ZJHFMPGASA-N
9-HOTrE	293.2 -> 171.1	(d4) 9(S)-HODE	9S-hydroxy-10E,12Z,15Z-octadecatrienoic acid	LMFA02000024	RIGGEAZDTKMXSI-MEBVTJQTSA-N
9-HpODE	311.2 -> 185.2	(d4) 9(S)-HODE	9S-hydroperoxy-10E,12Z-octadecadienoic acid	LMFA02000012	JGUNZIWGNMQSBM-UINYOVNOSA-N
9-KODE	293.2 -> 185.2	(d4) 9(S)-HODE	9-oxo-10E,12Z-octadecadienoic acid	LMFA02000274	LUZSWWYKKLTDHU-ZJHFMPGASA-N
Hepoxilin A3	335.2 -> 273.2	(d8) 12(S)-HETE	8-hydroxy-11S,12S-epoxy-5Z,14Z,9E-eicosatrienoic acid	LMFA03090005	SGTUOBURCVMACZ-SEVPPISGSA-N
LTB4	335.2 -> 195.1	(d4) LTB4	5S,12R-dihydroxy-6Z,8E,10E,14Z-eicosatetraenoic acid	LMFA03020001	VNYSSYRCGWBHLG-AMOLWHMGSA-N
LTB5	333.2 -> 195.1	(d4) LTB4	5S,12S-dihydroxy-6Z,8E,14Z,17Z-eicosapentanenoic acid	LMFA03020010	BISQPGCQOHLHQK-HDNPQISLSA-N
LTC4	624.3 -> 272.1	(d4) LTE4	5S-hydroxy-6R-(S-glutathionyl)-7E,9E,11Z,14Z-eicosatetraenoic acid	LMFA03020003	GWNVDXQDILPJIG-SWOVEEFNSA-N
LTD4	495.2 -> 177.1	(d4) LTE4	5S-hydroxy-6R-(S-cysteinylglycinyl)-7E,9E,11Z,14Z-eicosatetraenoic acid	LMFA03020077	YEESKJGWJFYOOK-LDDGIIIKSA-N
LTE4	438.2 -> 333.2	(d4) LTE4	5S-hydroxy,6R-(S-cysteinyl),7E,9E,11Z,14Z-eicosatetraenoic acid	LMFA03020002	OTZRAYGBFWZKMX-FRFVZSDQSA-N
Resolvin D1	375.2 -> 141.0	(d4) LTB4	7S,8R,17S-trihydroxy-4Z,9E,11E,13Z,15E,19Z-docosahexaenoic acid	LMFA04000006	OIWTWACQMDFHJG-CCFUIAGSSA-N

Table S5 Complete ROS/RNS pathway oxidised lipid target list

Metabolites	Precursor ion Product ion (*m/z*)	ISTD	Formal name	Lipidmaps ID	InChlKey
NO2-aLA (C18:3)	322.1 -> 46.05	10-Nitrooleate-d17	nitro-9E,12Z,15Z-octadecadienoic acid	*na*	*na*
NO2-LA (C18:2)	324.3 -> 277.25	10-Nitrooleate-d17	nitro-9E,12Z-octadecadienoic acid	LMFA01120001	LELVHAQTWXTCLY-XYWKCAQWSA-N
NO2-OA (C18:1)	326.1 -> 46.0	10-Nitrooleate-d17	nitro-9E-octadecenoic acid	LMFA01120003	WRADPCFZZWXOTI-BMRADRMJSA-N
10-HDoHE	343.2 -> 153.0	(d8) 12(S)-HETE	(+/-)-10-hydroxy-4Z,7Z,11E,13Z,16Z,19Z-docosahexaenoic acid	LMFA04000027	DDCYKEYDTGCKAS-SKSHMZPZSA-N
11-HDoHE	343.2 -> 121.0	(d8) 12(S)-HETE	(+/-)-11-hydroxy-4Z,7Z,9E,13Z,16Z,19Z-docosahexaenoic acid	LMFA04000028	LTERDCBCHFKFRI-BGKMTWLOSA-N
11-HETE	319.2 -> 167.1	(d8) 12(S)-HETE	11R-hydroxy-5Z,8Z,12E,14Z-eicosatetraenoic acid	LMFA03060028	GCZRCCHPLVMMJE-WXMXURGXSA-N
13-HDoHE	343.2 -> 281.0	(d8) 12(S)-HETE	(+/-)-13-hydroxy-4Z,7Z,10Z,14E,16Z,19Z-docosahexaenoic acid	LMFA04000029	SEVOKGDVLLIUMT-SKSHMZPZSA-N
14-HDoHE	343.2 -> 205.0	(d8) 12(S)-HETE	(+/-)-14-hydroxy-4Z,7Z,10Z,12E,16Z,19Z-docosahexaenoic acid	LMFA04000030	ZNEBXONKCYFJAF-BGKMTWLOSA-N
16-HDoHE	343.2 -> 233.0	(d8) 12(S)-HETE	(±)16-hydroxy-4Z,7Z,10Z,13Z,17E,19Z-docosahexaenoic acid	LMFA04000031	CSXQXWHAGLIFIH-VUARBJEWSA-N
17-HDoHE	343.2 -> 281.3	(d8) 12(S)-HETE	(±)17-hydroxy-4Z,7Z,10Z,13Z,15E,19Z-docosahexaenoic acid	LMFA04000032	SWTYBBUBEPPYCX-VIIQGJSXSA-N
18-HEPE	317.2 -> 255.0	(d8) 12(S)-HETE	(±)-18-hydroxy-5Z,8Z,11Z,14Z,16E-eicosapentaenoic acid	LMFA03070033	LRWYBGFSVUBWMO-UXNZXXPISA-N
2,3-dinor-8-iso-PGF2a	325.1 -> 237.2	(d4) 8-iso-PGF2a	9α,11α,15S-trihydroxy-2,3-dinor-(8β)-prosta-5Z,13E-dien-1-oic acid	LMFA03110010	IDKLJIUIJUVJNR-JSEKUSAISA-N
20-HDoHE	343.2 -> 299.0	(d8) 12(S)-HETE	(+/-)-20-hydroxy-4Z,7Z,10Z,13Z,16Z,18E-docosahexaenoic acid	LMFA04000033	YUZXOJOCNGKDNI-LFVREGEGSA-N
4-HDoHE	343.2 -> 281.0	(d8) 12(S)-HETE	(±)4-hydroxy-5E,7Z,10Z,13Z,16Z,19Z-docosahexaenoic acid	LMFA04000024	IFRKCNPQVIJFAQ-PQVBWYSWSA-N
(+/-) 5-iPF2a VI	353.3 -> 115.05	(d11) 5-iPF2a IV	5,9S,11R-trihydroxy-6E,14Z-prostadienoic acid-cyclo[8S,12R]	LMFA03110011	RZCPXIZGLPAGEV-SUHLLOIRSA-N
7-HDoHE	343.2 -> 281.0	(d8) 12(S)-HETE	(±)7-hydroxy-4Z,8E,10Z,13Z,16Z,19Z-docosahexaenoic acid	LMFA04000025	OZXAIGIRPOOJTI-XJAVJPOHSA-N
8,12-iPF2a VI	353.3 -> 115.05	(d11) 8,12-iPF2a IV	(±)5,9α-trihydroxy-12α-prosta-6E,14Z-dien-1-oic acid	*na*	RZCPXIZGLPAGEV-DCOIXEBESA-N
8_HDoHE	343.2 -> 189.0	(d8) 12(S)-HETE	(±)8-hydroxy-4Z,6E,10Z,13Z,16Z,19Z-docosahexaenoic acid	LMFA04000026	ZHBVYDMSPDDAKE-VTIZNUJUSA-N
8-HETE	319.2 -> 155.1	(d8) 5(S)-HETE	(±)8-hydroxy-5Z,9E,11Z,14Z-eicosatetraenoic acid	LMFA03060086	NLUNAYAEIJYXRB-HEJOTXCHSA-N
8-iso-13,14-dihydro-PGF2a	353.3 -> 183.1	(d4) 8-iso-PGF2a	9S,11R-dihydroxy-15-oxo-5Z-prostaenoic acid-cyclo[8S,12R]	LMFA03110004	VKTIONYPMSCHQI-JPRPWBOBSA-N
8-iso-15-keto-PGE2	349.1 -> 287.2	(d4) 8-iso-PGE2	9,15-dioxo-11α-hydroxy-(8β)-prosta-5Z,13E-1-oic acid	LMFA03110009	YRTJDWROBKPZNV-RSNVZYGJSA-N
8-iso-15-keto-PGF2a	351.1 -> 315.15	(d4) 8-iso-PGF2a	9α,11α-dihydroxy-15-oxo-(8β)-prosta-5Z,13E-dien-1-oic acid	LMFA03110005	LOLJEILMPWPILA-RLXHZABYSA-N
8-iso-15-keto-PGF2b	351.1 -> 315.15	(d4) 8-iso-PGF2a	9β,11α-dihydroxy-15-oxo-(8β)-prosta-5Z,13E-dien-1-oic acid	*na*	*na*

8-iso-15(R)-PGF2a	353.3 -> 193.2	(d4) 8-iso-PGF2a	9α,11α,15R-trihydroxy-(8β)-prosta-5Z,13E-dien-1-oic acid	LMFA03110030	PXGPLTODNUVGFL-PGWUFSIFSA-N
8-iso-PGA1	335.1 -> 273.15	(d4) PGA2	9-oxo-15S-hydroxy-(8β)-prosta-10,13E-dien-1-oic acid	LMFA03110008	BGKHCLZFGPIKKU-DRSVPBQLSA-N
8-iso-PGA2	333.1 -> 271.2	(d4) PGA2	9-oxo-15S-hydroxy-(8β)-prosta-5Z,10,13E-trien-1-oic acid	LMFA03110140	MYHXHCUNDDAEOZ-UKUWKSPLSA-N
8-iso-PGE1	353.3 -> 317.2	(d4) PGE1	9-oxo-11α,15S-dihydroxy-(8β)-prost-13E-en-1-oic acid	LMFA03110002	GMVPRGQOIOIIMI-JCPCGATGSA-N
8-iso-PGE2	351.1 -> 271.15	(d4) 8-iso-PGE2	9-oxo-11α,15S-dihydroxy-(8β)-prosta-5Z,13E-dien-1-oic acid	LMFA03110003	XEYBRNLFEZDVAW-CLQOMRTCSA-N
8-iso-PGF1a	355.3 -> 311.1	(d9) 8-iso-PGF1a	9α,11α,15S-trihydroxy-(8β)-prost-13E-en-1-oic acid	*na*	DZUXGQBLFALXCR-PUCCXBQTSA-N
8-iso-PGF2a (15-F2t-IsoP)	353.3 -> 193.2	(d4) 8-iso-PGF2a	9α,11α,15S-trihydroxy-(8β)-prosta-5Z,13E-dien-1-oic acid	LMFA03110001	PXGPLTODNUVGFL-NAPLMKITSA-N
8-iso-PGF3a	351.1 -> 307.15	(d4) 8-iso-PGF2a	9α,11α,15S-trihydroxy-(8β)-prosta-5Z,13E,17Z-dien-1-oic acid	*na*	*na*
9-HEPE	317.2 -> 149.0	(d8) 12(S)-HETE	(±)-9-hydroxy-5Z,7E,11Z,14Z,17Z-eicosapentaenoic acid	LMFA03070029	OXOPDAZWPWFJEW-IMCWFPBLSA-N
9-HETE	319.2 -> 167.1	(d8) 12(S)-HETE	(±)-9-hydroxy-5Z,7E,11Z,14Z-eicosatetraenoic acid	LMFA03060089	KATOYYZUTNAWSA-OIZRIKEUSA-N
iPF2a-iV	353.3 -> 127.1	(d4) iPFa-VI	*na*	*na*	MZYZWZXTHYCVHQ-QXCZDIPSSA-N

Table S6 The selected panel of compounds

Pathway	Metabolites	*m/z* Precursor ion → Product ion	ISTD
**COX**	13,14-dihydro-PGF2a	353.30 -> 183.10	(d4) PGF2a
	PGE1	353.30 -> 317.20	(d4) PGE1
	PGE2	351.10 -> 271.15	(d9) PGE2
	PGF2a	353.30 -> 193.20	(d4) PGF2a
**ROS**	8-iso-13,14-dihydro-PGF2a	353.30 -> 183.10	(d4) 8-iso-PGF2a
	8-iso-15(R)-PGF2a	353.30 -> 193.20	(d4) 8-iso-PGF2a
	8-iso-PGF2a	353.30 -> 193.20	(d4) 8-iso-PGF2a
**RNS**	NO2-LA (C18:2)	324.30 -> 277.25	10-Nitrooleate-d17
	NO2-OA (C18:1)	326.10 -> 46.00	10-Nitrooleate-d17

Table S7 The GUS blank effect compared to non-hydrolysed urine

GUS	8-iso-15(R)-PGF_{2a}	8-iso-13,14-dihydro-PGF_{2a}	8-iso-PGF_{2a}	PGE_2	PGE_1	PGF_{2a}	13,14-dihydro-PGF_{2a}	NO_2-linoleic acid	NO_2-oleic acid
H. pomatia	42.15	0.00	41.76	2.64	0.06	128.39	0.00	0.00	0.00
**Bovine liver**	0.00	0.00	21.18	0.05	0.05	16.12	0.00	0.00	0.00
E. coli	0.00	0.00	0.00	0.06	0.00	16.80	0.00	0.00	0.00

The GUS blank effect is calculated by: Metabolite response $_{\text{Enzyme blank}}$/ Metabolite response $_{\text{Urine}}$ ×100%

Table S8 RSDs of free and total oxidised lipids measured in RA patient urine

Compound name	RSD(%)	
	Total oxidised lipid	Free oxidised lipid
**8,12-iPF2a IV**	2.31	1.72
**12,13-DiHOME**	6.98	2.78
**9,10-DiHOME**	1.91	2.9
**iPF2a Unknown**	5.86	4.49
**PGF2a**	7.21	4.88
**2_3-dinor-8-iso-PGF2a**	12.36	5.49
**5-iPF2a IV**	2.8	5.71
**9,10-EpOME**	20.51	7.03
**PGA2**		7.31
**9-HODE**	5.04	7.37
**9,12,13-TriHOME**	9.95	7.71
**D17, 6-ketoPGF1a**	7.97	8.41
**8,9-DiHETrE**	4.43	8.64
**13-KODE**	9.12	9.12
**12-HETE**	9.35	9.4
**13-HODE**	7	9.96
**8-iso-PGF1a**	11.97	10.05
**PGF1a**	23.33	10.17
**20-carboxy-LTB4**	13.96	10.18
**14,15-DiHETrE**	4.24	11.04
**8-iso-PGF2a**	17.37	11.1
**9-HOTrE**	4.2	11.82
**5-iPF2a-VI**	11.46	11.85
**PGE1**		11.9
**9,10,13-TriHOME**	7.09	13.22
**8-iso-15(R)-PGF2a**	5.66	13.38
**12,13-EpOME**	32.38	13.8
**2,3-dinor-11b-PGF2a**	14.54	16.72
**13_14-dihydro-PGF2a**	13.49	18.19
**bicyclo-PGE2**	13.84	19.93
**14-HDoHE**	25.99	19.97
**PGE2**	11.63	20.86
**11beta-13,14-dihydro-15-keto-PGF2a**	16.83	20.97
**11beta-PGF2a**	21.92	23.49

**NO2-LA**		23.56
**8-iso-PGF2a**	23.8	23.85
**5S,6R-LipoxinA4**	14.72	25.29
**9-KODE**	25.86	25.6
**5S-HEPE**	6.66	26.29
**NO2-aLA**		26.33
**12S-HEPE**	19.08	26.57
**PGF2a**	9.49	26.83
**NO2-OA**		50.11
**8-iso-PGE1**		22.85
**19,20-DiHDPA**	5.1	
**9-HEPE**	6.96	
**15S-HETrE**	7.82	
**11,12-DiHETrE**	8.05	
**PGK2**	8.1	
**5,6-DiHETrE**	9.7	
**17,18-DiHETE**	10.24	
**12,13-DiHODE**	11.97	
**PGF3a**	12.63	
**20-HETE**	12.64	
**PGE3**	13.39	
**20-HDoHE**	14.14	
**5-HETE**	14.71	
**2_3-dinor-11b-PGF2a**	14.74	
**9-HETE**	14.86	
**iPF2a**	15.55	
**8-HETE**	16.58	
**11,12-EpETrE**	17	
**10-HDoHE**	17.14	
**15-HETE**	17.64	
**13,14-dihydro-15-keto-PGE2**	20.19	
**11-HDoHE**	20.44	
**11-HETE**	21.19	
**PGD3**	21.98	
**14,15-DiHETE**	22.37	
**8_HDoHE**	23.31	
**5S,15S-DiHETE**	23.9	
**16-HDoHE**	24.46	
**13,14-dihydro-15-keto-PGF2a**	41.36	

Table S9 Multiple linear regression models showing independent clinical parameters associated with oxidised lipid levels

Clinical parameter	Metabolite	Coefficient	*P* value
**CRP**	PGF3a	-0.28	0.08
	12-HETE	-0.32	0.06
	16-HDoHE	0.26	0.03
	20-HETE	0.19	0.08
	PGD3	-0.63	0.03
	PGE3	-0.55	0.05
**Baseline DAS28**	8-iso-PGF2a	0.19	0.06
	14,15-DiHETE	0.26	0.04
	14-HDoHE	-0.39	0.09
	5S,6R-LipoxinA4	0.67	0.01
	NO2-aLA	-0.47	0.02
	NO2-OA	-0.26	0.03
	NO2-LA	-0.48	0.00
**DAS28 improvement**	PGF3a	0.26	0.02
	PGF2a	0.11	0.02
	iPF2a	0.12	0.02
	NO2-LA	0.18	0.06
	11,12-EpETrE	0.15	0.04
	11-HETE	0.15	0.02
	13-KODE	0.18	0.08
	14,15-DiHETrE	0.10	0.02
	14-HDoHE	0.31	0.03
	15S-HETrE	0.14	0.05
	9-KODE	0.14	0.10
	bicyclo-PGE2	0.14	0.09
	NO2-LA	0.22	0.03

Figures

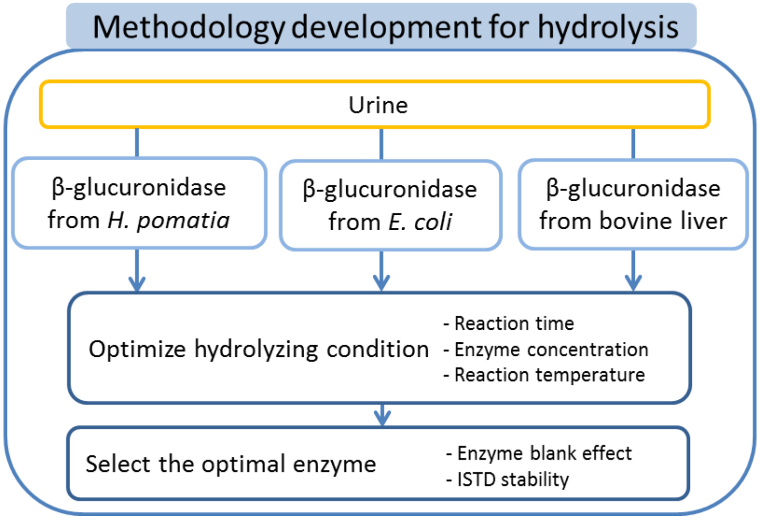

Fig. S1 Method development overview

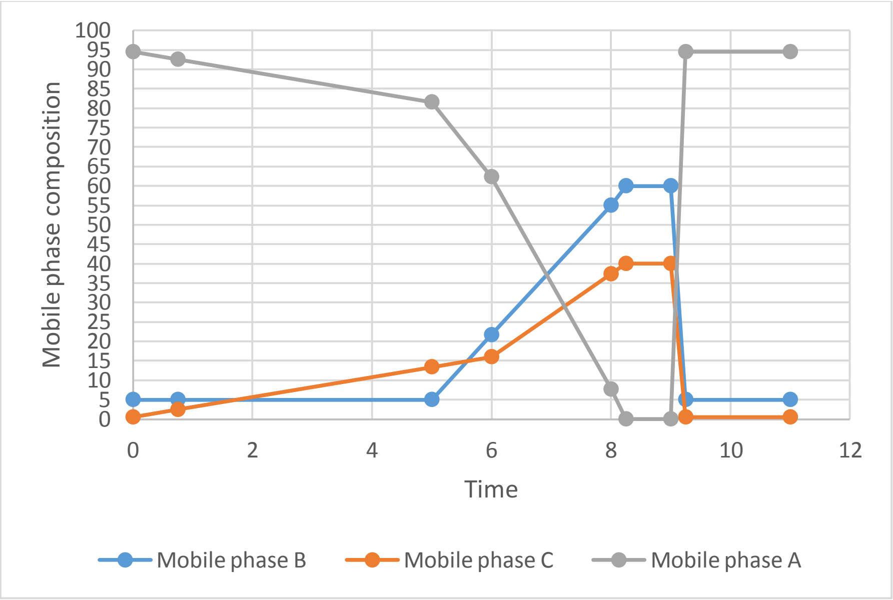

Fig. S2 Ternary gradient of the used UHPLC-MS/MS method

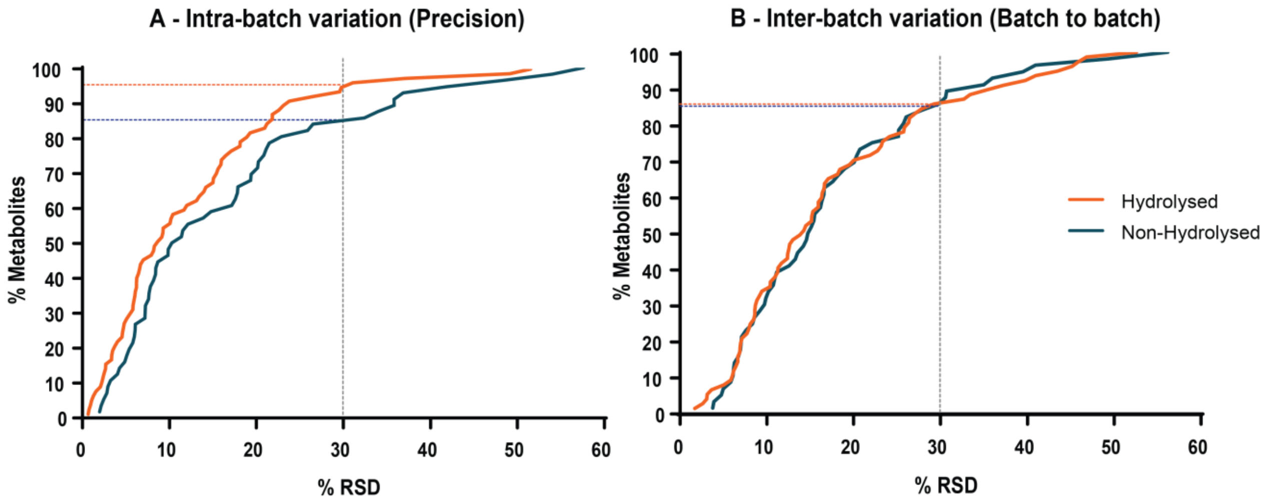

Fig. S3 Detected endogenous oxidised lipids evaluated for A - precision and B - batch-to-batch effect. More than 90% of endogenous metabolites had an RSD below 30% for the hydrolysed procedure's precision, followed closely with 85% of metabolites in the non-hydrolysed procedure having a precision below 30%. Inter-batch variation indicated similar stable performance across three measurement days for both the hydrolyses and non-hydrolyses procedure

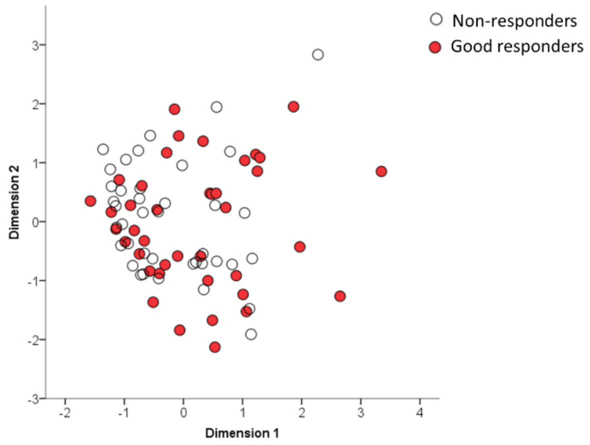

Fig. S4 Score plot of the CATPCA model for good and non-responders to TNF-α inhibitor treatment of RA patients, depicting component score on Dimension 1 and 2

Methods

Categorical principal components analysis

Categorical principal components analysis (CATPCA) is a nonlinear principal components analysis technique, which allows for different analysis levels (numeric, ordinal and nominal) for variables and is able to handle categorically and numerically measured variables[1, 2]. In our study, the variable "3-month response" (two categories: good responder, non-responder) was projected onto the model as a multiple nominal supplementary variable, which allows the observation of relationships between the therapeutic response and the other variables without interfering with the model.

1. Linting M, van der Kooij A (2012) Nonlinear Principal Components Analysis With CATPCA: A Tutorial. J Pers Assess 94:12–25. doi: 10.1080/00223891.2011.627965

2. Linting M, Meulman JJ, Groenen PJF, van der Koojj AJ (2007) Nonlinear principal components analysis: introduction and application. Psychol Methods 12:336–58. doi: 10.1037/1082-989X.12.3.336
